# Induced Vascular Normalization—Can One Force Tumors to Surrender to a Better Microenvironment?

**DOI:** 10.3390/pharmaceutics15082022

**Published:** 2023-07-26

**Authors:** Xu Xin Sun, Zeynab Nosrati, Janell Ko, Che-Min Lee, Kevin L. Bennewith, Marcel B. Bally

**Affiliations:** 1 Experimental Therapeutics, BC Cancer Research Institute, Vancouver, BC V5Z 1L3, Canada; znosrati@bccrc.ca (Z.N.); jko@bccrc.ca (J.K.); cmlee@bccrc.ca (C.-M.L.); kbennewi@bccrc.ca (K.L.B.); mbally@bccrc.ca (M.B.B.); 2 Interdisciplinary Oncology, BC Cancer Research Institute, Vancouver, BC V5Z 1L3, Canada; 3 NanoMedicines Innovation Network, Vancouver, BC V6T 1Z3, Canada; 4 Cuprous Pharmaceuticals, Vancouver, BC V6N 3P8, Canada; 5 Pathology & Laboratory Medicine, University of British Columbia, Vancouver, BC V6T 1Z4, Canada; 6 Faculty of Pharmaceutical Sciences, University of British Columbia, Vancouver, BC V6T 1Z4, Canada

**Keywords:** cancer angiogenesis, nanomedicine, tumor vascular normalization, immune cells, immunotherapy, drug repurposing, anti-angiogenesis, immune checkpoint inhibitors

## Abstract

Immunotherapy has changed the way many cancers are being treated. Researchers in the field of immunotherapy and tumor immunology are investigating similar questions: How can the positive benefits achieved with immunotherapies be enhanced? Can this be achieved through combinations with other agents and if so, which ones? In our view, there is an urgent need to improve immunotherapy to make further gains in the overall survival for those patients that should benefit from immunotherapy. While numerous different approaches are being considered, our team believes that drug delivery methods along with appropriately selected small-molecule drugs and drug candidates could help reach the goal of doubling the overall survival rate that is seen in some patients that are given immunotherapeutics. This review article is prepared to address how immunotherapies should be combined with a second treatment using an approach that could realize therapeutic gains 10 years from now. For context, an overview of immunotherapy and cancer angiogenesis is provided. The major targets in angiogenesis that have modulatory effects on the tumor microenvironment and immune cells are highlighted. A combination approach that, for us, has the greatest potential for success involves treatments that will normalize the tumor’s blood vessel structure and alter the immune microenvironment to support the action of immunotherapeutics. So, this is reviewed as well. Our focus is to provide an insight into some strategies that will engender vascular normalization that may be better than previously described approaches. The potential for drug delivery systems to promote tumor blood vessel normalization is considered.

## 1. Introduction

Immunotherapy is a cancer treatment that boosts a patient’s immune system to identify and destroy cancer cells. Immunotherapy has gained much attention over the past 15 years because it provides a new treatment approach for cancer patients. This would be in addition to treatments that involve surgery, chemotherapy, and radiation therapy [1]. Immunotherapies can be divided into passive and active treatments. Passive treatments include cytokine-based therapies and immune checkpoint inhibitors (ICIs), whereas active treatments encompass targeted antibodies, chimeric antigen receptor T cell (CAR-T cell), and dendritic cell-based cancer vaccines, approaches summarized in Table 1 [1,2].

The approved immune checkpoint inhibitors (ICIs) and cytokines provide benefits for patients with various solid tumors and blood cancers, but CAR-T cell therapy is currently limited to patients with leukemia and lymphomas [4,5]. Regardless of the immunotherapy approach used, there is a general understanding that the tumor microenvironment (TME) plays a significant role in treatment outcomes [6,7]. 

What is clear at this time is that the presence of tumor neoantigens and the TME play significant roles affecting the outcomes in patients receiving immunotherapeutics. There are three major components defining the TME in addition to the tumor cells and their neoantigens—(i) the tumor vasculature (TV), (ii) the tumor stroma, and (iii) the tumor-infiltrated immune cells—all of which are highly dynamic and heterogeneous [7]. All of the three components change over time as the tumor develops in defined locations and will differ dependent on the location(s) where the tumor grows [7]. It has been argued that changing the TME could change a tumor’s immune-suppressive environment into an immune-supportive environment. This change should increase progression free survival (PFS) and overall survival (OS) in patients that benefit from cancer immunotherapies [8,9].

This review has been organized to focus on the contribution of cancer angiogenesis during cancer development and how angiogenesis changes the TME. A detailed review of tumor vascular normalization (TVN) as an immunomodulatory strategy to improve immunotherapy outcomes is provided. In this context, our primary interest is to gain a better understanding of the evidence defining which small-molecular-weight drugs can engender TVN, how they can be administered to achieve that, as well as some speculation as to why TVN is achieved. Moreover, related to these points, this review aims to discuss how drug delivery systems (specifically liposomes and lipid nanoparticles) can be developed as nanomedicines that can augment the TVN effect.

## 2. Cancer Angiogenesis and the TME

Cancer angiogenesis is recognized as one of the cancer hallmarks, and studies on angiogenesis have led to the discovery of many factors and signalling pathways that could potentially target angiogenesis [10]. The initiation of this process is dependent on the binding and signalling of pro-angiogenic factors including vascular endothelial growth factor (VEGF) and basic fibroblast growth factor (bFGF) which are upregulated in response to inflammatory mediators [11]. However, before angiogenesis is initiated, an avascular phase exists where the tumor may remain dormant until the ‘angiogenic switch’ occurs. This occurs in the presence of proangiogenic factors, dominating over the effects of anti-angiogenic signals [12,13]. Proangiogenic factors include VEGF, bFGF, transforming growth factor-β (TGF-β), and interleukin 8 (IL-8), while anti-angiogenic factors include angiostatin, endostatin, vasostatin, and interleukin 12 (IL-12) [12,14]. It is believed that many immune cells, during this avascular phase, begin to gradually respond and aggravate the suppression effect on the immune system which eventually results in cancer immune evasion [15]. For example, the accumulation of TGF-β greatly inhibits the maturation of dendritic cells and effector T cells and stimulates the recruitment of regulatory T cells (Treg cells) [16]. The expansion of Tregs inhibits cytotoxic T cell functions and polarizes macrophages to the pro-tumor M2 type [16,17].

### 2.1. Role of Hypoxia in Tumor Progression

One of the triggers leading to the production of pro-angiogenic factors is tumor hypoxia. The evolving tumor requires more oxygen and nutrients than what is available through normal but distant blood vessels. This leads to the formation of areas of hypoxia [18]. Hypoxia signalling is mediated by the evolutionarily conserved hypoxia-inducible factor (HIF) pathway that instantly responds to low oxygen in the environment to promote angiogenesis and tumor cell migration. The HIF pathway also encourages tumor growth by inducing the gene expression of pro-angiogenic factors including VEGF and angiopoietin-2 (Ang-2) [19,20]. HIF signalling is contingent on HIF-1α, which is constitutively expressed. In normoxic conditions, HIF-1α is proteolytically degraded but when produced in a hypoxic environment, HIF-1α becomes stabilized. Stabilized HIF-1α binds its partner HIF-1β and translocates to the nucleus triggering a cascade of downstream signalling to mitigate hypoxia-mediated death, preserve metabolites the tumor cells may need, and promote tumor cell migration so the cells can escape from the area of hypoxia [21,22]. Two forms of tumor hypoxia exist—chronic and cycling hypoxia [23]. Chronic (diffusion-limited) hypoxia prevents oxygen from diffusing into cells that have been pushed too far away (>70–100 μm) from blood vessels due to the proliferation of tumor cells [24]. As these cells experience hypoxia, their ability to proliferate diminishes, and they start to localize in areas of necrosis [25]. Cycling or transient (perfusion-limited) hypoxia occurs when blood flow is suddenly halted for a varying degree of time [23]. As transiently hypoxic tumor cells still undergo HIF-1-induced gene expression changes, including increases in migration-related genes, these cells are poised to move into the blood vessel at the moment when functional blood vessel perfusion occurs [25,26]. Thus, transient hypoxia in the tumor is of concern for tumor cell migration and metastasis, and normalizing TV is thought to prevent the development of transient hypoxia.

### 2.2. Role of Hypoxia in Tumor Immune Suppression

Under hypoxic conditions, HIF signalling shifts the glucose metabolism from oxidative phosphorylation to glycolysis [20,27]. When cancer cells are rapidly dividing, even in the presence of O_2_, the HIF-1α subunit also serves as one of the mediators for cells to preferentially utilize aerobic glycolysis as their energy source [27]. The metabolic changes are accompanied by the secretion of lactic acid, decreasing the local pH [15,27]. As one might expect, low pH is generally detrimental to cells in the TME, but it is worth noting in a typical inflammatory environment some immune cells sense protons through proton receptors which can activate the nuclear factor kappa-light-chain-enhancer of activated B cells (NF-κB). NF-κB can then activate an innate and adaptive pro-inflammatory immune response [28,29]. The proliferation and function of most immune cells is not only dependent on the pH but also cell surface receptors, such as chemokine receptors, and oxygen levels [27]. In a solid tumor, combining the tumor hypoxia and the decrease in local pH, many published studies suggest there are devastating effects from lactic acid and hypoxia on various immune cell populations [15,30,31].

Starting with monocytes, extracellular acidosis suppresses the expression of monocyte chemoattractant protein-1 (MCP1) and IL-6, both of which are critical for the maintenance of the pro-inflammatory M1 macrophage phenotype in the TME while at the same time promoting M1 to tumor-associated macrophage (TAM/M2) subtype transformation by increasing mannose receptor C-type 1 and arginase 1 expression [31,32]. The acidic environment also stimulates macrophages to produce IL-1β, which usually predicts a poor prognosis in many cancer types [32,33]. Monocytes lose their ability to acquire the cluster of differentiation 1α expression necessary for the differentiation into monocyte-derived dendritic cells (DCs). Consequently, this results in the reduction in the ability of DCs to produce the key anti-tumor cytokine IL-12 [32].

Cytotoxic T cells (CTLs) are also sensitive to the external lactic acid concentration. When external pH is decreased by metabolic changes in the tumor cells, the cytotoxic functions of CTLs are disrupted through the blockade of monocarboxylate transporter-1 [34,35]. Although this immunosuppressive effect on T cell functions is thought to be reversible, the lactic acidosis and hypoxia together decrease the infiltration of both CD4+ T helper cells and CD8+ cytotoxic T cells [34,35,36]. In addition to this, hypoxia along with the VEGF-A are demonstrated to cause CD8+ T cell exhaustion, associated with the differentiation into a terminal state (PD-1^+^ TIM-3^+^ CXCR5^+^) [37]. While in many studies HIF-1α was demonstrated to act negatively on T cell functions, other studies revealed that HIF-1α can be important in balancing the proliferation of pro-inflammatory Th17 cells and the regulatory T (Treg) cell population [38].

Various mechanisms explaining how immune cells sense the pH and oxygen within the TME have been suggested, some of them contrast to what is observed in typical sites of inflammation. Whether this pH sensing process (decreased pH downregulating the immune cells’ anti-tumor functionality in general) is proton receptor dependent or independent remains poorly understood [32]. However, it can be argued that targeting tumor-associated angiogenesis and normalizing the vasculature in tumors (alleviating the hypoxic and acidic conditions within a tumor) will be beneficial to an anti-tumor immune response.

### 2.3. Major Targets in Cancer Angiogenesis That Have Immunomodulatory Effects

The proliferation and migration of endothelial cells are mainly regulated by the combined activity of three major growth factors binding to their respective receptors—VEGF, bFGF, and platelet-derived growth factor (PDGF) [39]. Different from VEGF and bFGF, which act directly on endothelial cells, PDGF, in a hypoxic environment in the tumor, indirectly promotes angiogenesis by regulating the VEGF mRNA expression [40]. Over the past decade, studies have suggested that targeting these factors displays a strong immunomodulatory effect. Such an effect is most prominent when several factors can be inhibited simultaneously [39,41]. Most anti-angiogenic therapy treatments target the VEGF signalling pathway, but as pre-clinical results suggest, targeting the FGF and PDGF signalling pathways could be used in combination in the development of new anti-angiogenic therapies [41,42].

#### 2.3.1. VEGF

VEGF is the most essential pro-angiogenic factor of angiogenesis. In the presence of hypoxic conditions and certain growth factors including epidermal growth factor (EGF) and TGF-β, VEGF is upregulated and binds to vascular endothelial growth factor receptor-1 (VEGFR-1) or receptor-2 (VEGFR-2) on endothelial cells to promote new endothelial cell growth and proliferation [30,43]. The interaction between VEGF and the angiopoietin-2 (Ang-2)/Tie signalling system promotes the dissolution of the basement membrane by proteases, thereby increasing the “leakiness” of tumor-associated blood vessels [44]. Ang-2 is highly immunosuppressive and is usually elevated in a pre-metastatic niche and has been proven to support tumor cell extravasation in early-stage metastases [45]. It suppresses CTL function by recruiting Tie-expressing monocytes, a mechanism that the primary tumor uses both locally and as a systemic signal to tumor cells that metastasized to distant organs [45,46]. Therefore, agents that inhibit VEGF and/or block the Ang-2/Tie pathway have been widely used in the clinic to support immunotherapies, such as ICIs [47].

#### 2.3.2. bFGF

bFGF/FGF-2 is another proangiogenic factor that plays a key role in vascular endothelium integrity [43]. Basic FGF promotes the migration of endothelial cells and extracellular matrix (ECM) degradation by increasing the production of plasminogen activator and collagenase to weaken the endothelium’s basement membrane [13,30]. FGF binds its receptors on endothelial cells which activates the intrinsic tyrosine kinase and induces the transformation of normal endothelial cells into tumor-associated endothelial cells. This transition causes the blood vessels to increase their permeability because cells are now actively proliferating and migrating [48]. It is thought that bFGF primarily acts on TAMs, and by deleting FGF-2, macrophages could be re-polarized to the iNOS^+^/CD206^+^ anti-tumor M1 phenotype [49].

#### 2.3.3. PDGF

PDGF-BB is the most active isoform in the PDGF family and is involved in the recruitment and differentiation of pericytes and vascular smooth muscle cells during vascularization [43,50]. Studies show that pericytes can prevent the inhibition of VEGF signalling on endothelial cells, suggesting that the combination of PDGF and VEGF antagonists may enhance anti-angiogenic therapies [41,42]. Additionally, studies have also shown that FGF-2 and PDGF-BB act synergistically to stimulate angiogenesis. FGF-2 was found to be responsible for the upregulation of the PDGF receptor expression in endothelial cells, while PDGF-BB can promote FGF receptor-1 activity in vascular smooth muscle cells [50]. PDGF-BB is a major regulator of T cell proliferation (primarily CD4+ T cells) and activity, suppressing IL-4, IL-5, and interferon-γ (IFN-γ) secretion [51,52]. In response to the increases in PDGF-BB level, the level of pro-inflammatory cytokines IL-6, IL-8, CCL2, and CCL5 in the blood are negatively impacted [53,54].

## 3. TVN and Its Immunomodulatory Benefits

**Figure 1:**
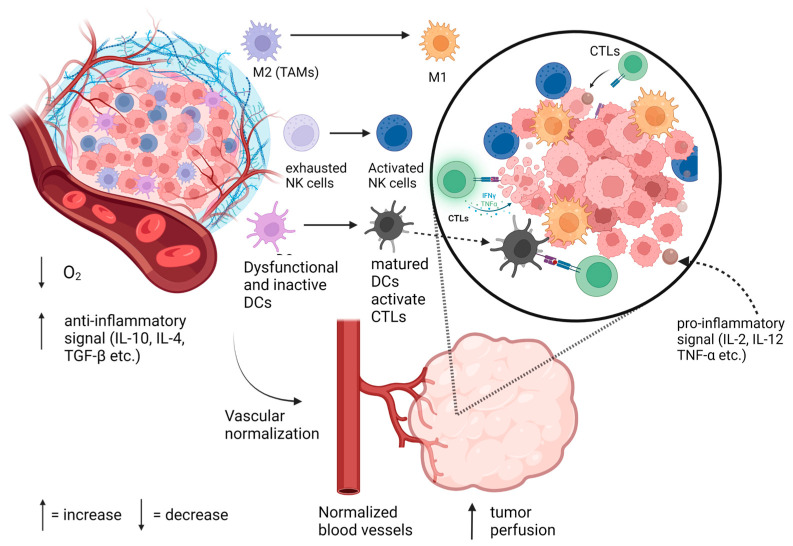
Immune cell population changes following tumor vascular normalization. The TME is hypoxic with increased levels of anti-inflammatory signals. TVN induces the polarization of TAM/M2 towards a pro-inflammatory type 1 macrophage (M1) type, reactivates exhausted natural killer cells (NK cells), and promote the maturation and activation of dendritic cells (DCs) in the tumor core. Further, there is infiltration and activation of CD8+ cytotoxic T cells (CTLs). More pro-inflammatory cytokines will be present in the TME, augmenting the immune response in the TME. Created with BioRender.com (accessed on 19 May 2023).

It was often assumed that anti-angiogenic treatments can destroy existing tumor blood vessels that overexpress VEGF and can block new tumor vasculature formation, a concept that would be comparable to cutting off the blood supply to the tumor [55]. However, Jain et al. and others changed this perspective, highlighting the concept of TVN [8,56]. The TVN process is associated with a change in the tumor’s vasculature from abnormal, leaky, and immature to a more balanced and functional “normal” tumor vasculature. The normalized blood vessel structures more effectively deliver oxygen and nutrients, as well as promoting immune cell infiltration into the tumor [8,57]. By stabilizing oxygen delivery to tumor cells, there will be less transient hypoxia development in the tumor, preventing the HIF-1-mediated upregulation of migration-related genes in the tumor cells. One way to demonstrate the TVN effect is by analyzing the CD31+ cell population (as a marker for microvessel density) in the tumor. Usually, it is expected that the percentage of CD31+ cells in the tumor would decrease [58,59,60]. When TVN is promoted, the tumor interstitial fluid pressure (IFP) decreases, and the TV is “repaired”. The normalized blood vessels have more complete coverage of vascular pericytes that facilitate not only the migration of immune effector cells but also influence the functions of these cells [61,62,63]. This TVN effect is normally accompanied by a significant reduction in pro-angiogenic and anti-inflammatory factors [64,65]. Multiple immune cell types, augmenting innate and adaptive immunity, are known to be enhanced by TVN, as illustrated in Figure 1. TVN can decrease hypoxia, enhance immune cell migration into the tumor, and convert the immunogenically “cold” tumor into a “hot” one. As proposed here and elsewhere, treatments that engender TVN should prove to be synergistic when used in combination with immunotherapies [64,66].

When considering the immune cells involved, there has been a focus on T cells, macrophages, dendritic cells, and natural killer (NK) cells. The presence or absence of these cells, their subtypes, and their functions help to differentiate a “cold” tumor from a “hot” tumor [67].

### 3.1. TVN and T Cells

Melanoma is one of the most well-known “cold” hypoxic tumor types with few immune cells [66]. In a recent study conducted using the B16F10 murine melanoma model, Chelvanambi et al. reported that a low dose of the stimulator of interferon gene (STING) agonist ADU S-100 induced TVN and subsequently increased the infiltration of CD8^+^ T cells and CD11c^+^ DCs, converting the immunological “cold” TME into a “hot” TME [68]. Zhang et al. showed that disrupting VEGF expression using Delta-like-1-factor successfullyinduced TVN, increased the total number of cytotoxic CD8+ T cells in an EO771 murine breast cancer model and was synergetic with anti-CTLA-4 mAbs [69]. Not only anti-angiogenic agents could improve the TVN effect, resulting in improved overall T cell activities. A small-molecule drug CU06-1004 altered blood vessel permeability and promoted CD8+ T cell proliferation and cancer cell killing when combined with anti-PD-1 inhibitors [70]. Interestingly, the disruption of the VEGF/VEGFR signalling pathway alone sometimes appears insufficient and could be even detrimental. In a study using a hepatocellular carcinoma model, tumor-infiltrating CD4+ T cells responded to a VEGFR-2 blockade by increasing the PD-1 expression levels and further inhibiting CTL function. However, upon the addition of anti-PD-1 antibodies, TVN was promoted [71]. Researchers also noted the depletion of regulatory T (Treg) cells (a pro-tumoral CD4+ T cell subtype) when there was a simultaneous inhibition of PD-1 and VEGFR-2, which was not typically achievable by ICI therapies alone [72,73,74]. The results revealed the crucial role of CD4+ T cells in maintaining the TME and in expanding immature TV. The presence of Treg cells in some tumors resulted in the upregulation of CCL28. CCL28 upregulation is under the control of HIF-1α [75], and the alleviation of hypoxia by TVN will reduce HIF-1α, abrogating CCL28 tumor-promoting effects, which leads to the inhibition of tumor growth [64,75].

### 3.2. TVN and Tumor-Associated Macrophages

Besides Treg cells, TAMs are another important mediator that are thought to contribute to tumor growth and a poor immunotherapy treatment response [76]. In a 4T1 mouse model of triple negative breast cancer (TNBC), TVN was achievedfollowing a treatment with a novel integrin-binding peptide.This treatment reduced the overall PD-L1 expression in the tumor including the PD-L1 expression on TAMs [77]. Also, in glioblastoma and colon cancer models, in response to Ang-2/VEGF inhibition, the treatment resulted in M2-macrophages being re-polarized to pro-inflammatory M1-subtype macrophages [78,79]. On the other hand, targeting TAMs can benefit normal blood vessel formation in the tumor. For example, a melittin-containing apoptosis-inducing peptide (MEL-dKLA) designed by Lee et al. demonstrated promising therapeutic effects in the Lewis lung carcinoma model by the specific depletion of M2-like TAMs (without impacting other leukocytes), resulting in an increase in the M1/M2 subtype ratio. The change in the TV was associated with a decrease in CD31+ cells. As a result, significant delayed tumor growth and prolonged survival were noted [80]. A similar TVN approach was achieved using histidine-rich glycoprotein (HRG), which also increased the M1/M2 subtype ratio [81].

### 3.3. TVN and Dendritic Cells and Myeloid-Derived Suppressor Cells

High levels of VEGF expression can affect the antigen-presenting ability and maturation of DCs in vitro and can promote the recruitment of myeloid-derived suppressor cells (MDSCs) in vivo [82,83]. When combined with high levels of anti-inflammatory cytokines, DCs will often express more immune checkpoint receptors including CTLA-4/CD80, PD-L1, and lymphocyte activation gene 3 on their surface, and this appears to limit immunotherapy outcomes, especially those that rely on T cell functions [84]. In this context, anti-VEGF therapy can decrease the infiltration of MDSCs and stimulate resident DC differentiation and the subsequent activation of Th1 helper T cells and CTLs. In aggregate, this resulted in enhanced anti-tumor activities [85]. In a clinical study, the normalization of TV in TNBC patients also led to mature DC infiltration [86]. In recent years, tuning DC functions and priming these cells as part of an immunotherapeutic regiment has defined a new way to fight cancer. The efforts led to the first FDA-approved dendritic cell vaccine for prostate cancer [82,84,87,88]. Though lacking direct evidence of the synergetic effect between a DC vaccine and TVN in clinical trials, the efforts of combining a cancer vaccine (cell or antigen based) with a vascular normalizing treatment in pre-clinical studies have proven successful in multiple cancer models [89,90,91].

### 3.4. TVN and Natural Killer Cells

While strategies to improve the outcomes of immunotherapy have been focused on the resuscitation of exhausted and suppressed CTL populations, TVN has been shown to rejuvenate NK cells. Yinli and colleagues found in a syngeneic mouse model of hepatocellular carcinoma (HCC) that treatment with apatinib (VEGRR-2 inhibitor) induced TVN. This was associated with decreases in tumor growth and the promotion of NK cell (CD3^−^NK1.1^+^) infiltration [92]. This was observed without any changes to CD4+ and CD8+ T cells in the tumor. Furthermore, the infiltrated NK cells were activated and expressed high levels of surface activation markers NKG2D and CD69 [92]. Like other immune cells that are suppressed by the TME, mechanisms that inhibit NK cell activity are postulated to be due to HIF-1α expression, which will decrease under a situation where tumor vascular normalization is achieved [93,94,95].

## 4. Anti-Cancer Treatments That Engender Tumor Vascular Normalization

As highlighted in Section 3, there is excellent justification for augmenting immunotherapy results by combining these treatments with strategies that promote vascular normalization. There have been many reviews that comprehensively examined the advantages and disadvantages of conventional anti-angiogenic therapies (i.e., anti-VEGF mAbs) [65,96]. The aim in this section is to focus on other strategies that have been investigated to promote TVN. Three are considered here: repurposing cardiovascular drugs that remodel the TME, metronomic dosing, and nanomedicines, specifically, lipid-based nanoparticles that deliver associated drug(s) in a manner that mimics metronomic dosing and/or could provide an improved method to deliver tumor vasculature normalization agents.

### 4.1. Induction of Tumor Vascular Normalization by Repurposing Cardiovascular Drugs

Certain regulatory pathways that are targets for cardiovascular disease treatment are also closely related to the pathways that define targets for cancer. It is therefore not surprising that certain cardiovascular drugs may be useful if repurposed for the treatment of cancer, particularly the treatments that use immunotherapeutics [97]. The repurposing of approved cardiovascular drugs for use in the treatment of cancer has greatly shortened the developmental time, in part because they are safe, have known side effects, and are well tolerated in humans. This strategy has proven to be very interesting if considered in combination with ICIs. In particular, in some studies, the results have already proven the benefits when using these drugs for the treatment of patients with different solid tumors, including bladder, colorectal, lung, breast, and melanoma cancers [98]. Several of the agents used in this context are summarized in Table 2.

#### 4.1.1. Renin Angiotensin Aldosterone System Inhibitors—ARBs and ACE-Is

The renin angiotensin aldosterone system (RAAS) is the master regulator of blood pressure in the body, with the peptide hormone angiotensin II (Angt II) being an important effector of the system. Angt II increases blood pressure by binding Angt II receptor type 1 (AT1R), which is expressed on various cells throughout the body. There are two main classes of drugs on the market that target RAAS to decrease blood pressure, either by directly competing with Angt II binding to AT1R (AT1R blockers: ARBs) or by inhibiting the angiotensin-converting enzyme (ACE) to prevent the production of Angt II. ARBs and angiotensin-converting enzyme inhibitors (ACE-Is) are gaining more attention for their potential to create TVN as a way to treat cancer [111]. In some retrospective studies, researchers found that cancer patients that were using previously prescribed ARB or ACE-I medication while receiving standard cancer treatments including chemotherapy, radiotherapy, or ICIs had better PFS and OS across various cancer types [112,113]. As a consequence of Angt II inhibition, either indirectly or directly (respectively), both ARBs and ACE-Is downregulate the expression of VEGF [97,114]. Previous studies have also suggested the role of a localized Angt II/AT1R axis in tumor growth, promoting immunosuppression within the tumor [99]. AT1R signalling can induce tumor hypoxia in the TME through the creation of ROS and/or by contributing to the physical barriers of the ECM, both of which hinder the efficacy of ICIs [115,116]. The immunosuppressive state of the TME can be improved by decreasing the levels of Angt II or blocking its activity with ARBs and/or ACE-Is which better support the dendritic cell maturation and T cell functions [97,99,117]. This, in turn, should enhance the effects of ICIs in tumors, especially for the tumors that have a high expression of angiotensin receptors [99]. ARBs and ACE-Is have been shown to have anti-angiogenic and immunomodulatory properties that can help modulate the vasculature within a tumor [97]. They can cause changes in the TME through effects on the tumor stroma. For example, hypoxia can induce fibrotic stroma in the TME, and this stroma interferes with the activity of immune cells and increases the expression of PD-L1 [98,99,114]. ARBs and ACE-Is modulate NF-κB and HIF-1α, which inhibits matrix metalloproteinases and decreases the expression of VEGF, also leading to an improved TV [97,98].

The efficacy of ARBs and ACE-Is is highlighted by several pre-clinical studies, notably for ARBs. Wadsworth et al. investigated the ARB telmisartan, which was found to alter the solid TME through reducing the activation of cancer-associated fibroblasts (CAFs) and collagen I deposition, improving tumor vascular perfusion and decreasing hypoxia, thereby improving the tumor’s response to radiation [117,118]. Telmisartan is also an attractive agent due to its improved bioavailability and strong affinity for AT1 receptors as compared to other ARBs [117,119]. Kosugi et al. showed that the ARB candesartan was able to decrease the expression of VEGF, inhibit angiogenesis, and suppress tumor growth in a mouse bladder cancer xenograft model [100]. In E0771, 4T1, and MCa-M3C breast cancer models, Chauhan et al. found an increased response rate to anti-PD-1 and anti-CTLA4 ICIs when they were combined with an ARB (valsartan) linked to a pH-sensitive polyacetal polymer [98]. Based on the amount of pre-clinical and retrospective clinical evidence, more clinical trials have been started using ARBs and ACE-Is as TVN-inducing agents to enhance immunotherapies. For example, a Phase II trial initiated in 2018 was designed to investigate the use of the angiotensin receptor blocker (ARB) losartan in combination with anti-PD-1 nivolumab and FOLFIRINOX for the treatment of pancreatic cancer (clinical trial number NCT03563248) [112].

#### 4.1.2. Beta-Blockers (β-Blockers)

It has been suggested that reducing physiological stress modulated by beta-adrenergic signalling can improve T-cell-dependent anti-tumor immune responses, and therefore, agents that block beta-adrenergic signalling could increase the efficacy of ICIs [102]. Norepinephrine released from the sympathetic nerve terminals is one of the main drivers of physiological stress responses. Norepinephrine acts by binding beta-adrenoceptors (β-AR) which are prominent in several cancer types including breast, pancreatic, and ovarian cancers [97,101]. While there are three types of β-AR subtypes (β1, β2, and β3), it appears that the binding of the β2 subtype is predominantly responsible for potential anti-tumor activities [102]. As part of the stress response, norepinephrine favours the accumulation of immunosuppressive cells in tumors, including myeloid-derived suppressor cells (MDSCs) and M2 macrophages. Further, this stress response is associated with inhibiting phagocytosis by macrophages and impairing the cytotoxicity of NK cells [98,103,120]. Thus, antagonists of β-ARs could provide benefits in the context of cancer by blocking the effects of norepinephrine to improve immune responses and the efficacy of ICIs. Retrospective studies showed that β-blockers increased survival rates in patients with malignant melanoma, breast cancer, epithelial ovarian cancer, and colorectal cancer [104]. Further, they are considered as a safer alternative to anti-angiogenic therapies (anti-VEGF mAbs) [102,104].

As norepinephrine decreases the production of IL-2, which is required for the proliferation of T cells, combining a β-blocker to block the effects of norepinephrine with IL-2 therapy may favour T-cell-dependent immunotherapy treatments [102]. Wrobel et al. observed a decrease in tumor vessel density and melanoma cell survival in a human xenograft melanoma model after treatment with the non-selective β-blocker propranolol [103]. Further, in a murine melanoma model, Kokolus et al. found that the β-blockers metoprolol and propranolol combined with a high-dose IL-2 therapy to increase the effectiveness of anti-PD-1 therapy [102]. Propranolol entered a Phase I clinical trial with an anti-PD-1 mAb (pembrolizumab) for the treatment of melanoma (clinical trial number NCT03384836). The combination was considered safe, and 78% of the patients responded with an increase of IFN-γ level in the blood, which was considered slightly better than what would have been expected with an anti-PD-1 mAb monotherapy alone [121]. Therefore, this combination might yield a positive outcome in the following Phase II trial (ongoing) and achieve synergistic anti-tumor activity in patients with unresectable stage III metastatic melanoma [121].

#### 4.1.3. Cyclooxygenase (COX) Inhibitors

COX inhibitors are another class of drugs used for the treatment and management of cardiovascular conditions that show promise if used in combination with ICIs and other cancer immunotherapeutics. The mechanism of action of this drug class involves the inhibition of prostaglandin synthesis through the inhibition of the COX enzyme [106]. The immunosuppression within the TME may be due to cyclooxygenase-2 (COX2)-induced prostaglandin E2 (PGE2) production [99,101]. This is thought to lead to immunotherapy resistance [99]. Further, COX2 overexpression is usually associated with a poor prognosis for many cancer types [105]. PGE2 is a major factor in the inflammatory response as it induces angiogenesis through the increasing expression of VEGF, and it can promote the immune evasion of cancer cells. Increased levels of PGE2 may be involved in the recruitment and accumulation of MDSCs which have strong immunosuppressive effects, including the inhibition of the immune activity of T cells and natural killer (NK) cells [105,107]. Additionally, PGE2 is involved in the activation of the indoleamine 2,3 dioxygenase pathway which depletes tryptophan, an essential amino acid which contributes to the survival of T effector cells in tumors [122,123]. Thus, the inhibition of COX2 may improve the outcomes of patients treated with ICIs by decreasing the expression of immunosuppressive factors such as IL-6 and IL-10 and increasing the expression of anti-tumor immune mediators such as IFN-γ and TNF-α [124,125,126].

Aspirin is perhaps the most used COX2 inhibitor. Ma et al. found that the use of a polymer-linked aspirin molecule was able to increase the infiltration of CD3+CD8+ and the M1/M2 macrophage ratio in a CT26 murine xenograft model [126]. Additionally, a decrease in MDSC and regulatory T cell infiltration was observed with the polymer-linked aspirin-treated group, converting the model from an immune suppressive environment to a to an immune supportive environment [126]. Several Phase II clinical trials have been initiated to understand the effects of COX2 inhibition in patients being treated with ICIs (such as NCT03396952 (began January 2018) and NCT03638297 (began June 2018)).

#### 4.1.4. Cardiac Glycosides (CGs)

CG cardiovascular drugs may potentially act as immunotherapeutic agents through their ability to exert ICD. [127] CGs are typically used for the treatment of congestive heart failure and cardiac arrhythmias by enhancing the contractile force (strength) of the heart [108]. These drugs can induce ICD through the inhibition of the Na/K-ATPase pump, which leads to the accumulation of intracellular Ca^2+^ and the translocation of CRT to the cell surface, causing secretion of ATP and HMGB1 [110,127]. Additionally, CGs were found to play a role in the modulation of FGF-2 and NF-κB [108]. Further, Li et al. obtained data to suggest that the CG oleandrin was able to increase the activation and infiltration of DCs and T cells into the EMT-6 murine breast cancer model [109]. Oleandrin was also shown to decrease the immunosuppressive factor IL-10 while increasing the secretion of the immune supportive factors IL-2 and IFNγ [109]. Although most clinical trials that repurpose CGs as potential anti-cancer agents are still at their early stages (pre-clinical/Phase I), they appear to be safe when administered with a wide range of immunotherapeutics and chemotherapies [128].

So, while it is easy to test drugs that are already approved for use in cardio vasculature disease in patients receiving ICIs, it is unclear whether the doses of these drugs used to treat cardiovascular disease are appropriate for the treatments of patients with cancer. Thus, when considering repurposing these drugs, it is important to consider the dose being used as well as the route and method of administration. As an alternative, our team is considering the use of drug carrier systems given intravenously for ARBs typically given orally.

### 4.2. Metronomic Dosing of Chemotherapy Drugs

**Figure 2:**
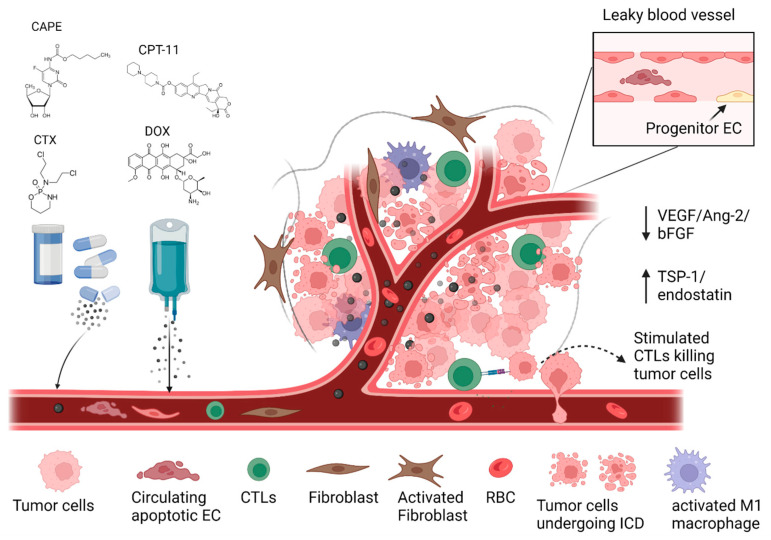
Metronomic chemotherapy changes the TME. Adapted from “The Tumor Microenvironment: Overview of Cancer-Associated Changes” by BioRender.com (2023). Retrieved from https://app.biorender.com/biorender-templates (accessed on 19 May 2023).

As indicated already, initial efforts to achieve TVN focused on the direct inhibition of the VEGF/Ang-2/VEGFR signalling pathway, and the therapeutic agents were typically administered at their maximum tolerated dose [129]. Perhaps surprisingly, investigators discovered that TVN could also be achieved by the metronomic dosing of chemotherapy drugs and radiation [64]. Even with anti-angiogenic therapies, metronomic dosing methods may be more effective at inducing TVN, modulating the TME, and improving OS in some aggressive tumors like glioblastoma [55,130,131]. Despite some concerns about metronomic chemotherapy (MC), such as the potential for the normalized vasculature to improve nutrient and oxygen delivery to tumor cells and enhancing tumor metastasis, the results from some clinical studies have shown the therapeutic benefits of MC. These benefits may be a result of changes in the TME and the use of MC in combination with other therapeutic modalities [132]. The potential benefits are illustrated in Figure 2. When considering the effects of MC, one must contemplate the direct cytotoxic (cell-killing) effects of MC on proliferating tumor endothelial cells (ECs), circulating ECs, and the inhibition of progenitor EC migration [133,134]. Further, the balance between pro-angiogenic (VEGF/VEGF-2/bFGF) factors and anti-angiogenic (TSP-1/endostatin) factors appears to be restored with MC [133]. In the context of immunotherapy, some of the drugs that exhibit improved activity when given metronomically, such as oxaliplatin (OXP), doxorubicin (DOX), and cyclophosphamide (CTX), are also known to promote ICD, and this may further enhance the effects of immunotherapies as mentioned previously [135,136].

ICD is a specific cell-death pathway that triggers an immune response, often characterized by the secretion of damage-associated molecular patterns (DAMPs). Three DAMPs that are considered indicative of ICD are ATP, high-mobility group box 1 (HMGB-1), and calreticulin (CRT) [137,138]. In tumors that have an immunosuppressive TME and lack the infiltration of immune effector cells, they could become more immunogenic if ICD is induced at the same time that tumor vasculature normalization is achieved, promoting the right immune effector cells to be present as discussed in the previous section [139]. Thus, the TVN effect of MC potentially creates a therapeutic window due in part to the alleviation of transient hypoxia, as well as the other effects noted. Together, these effects combine to maximize the synergistic immunomodulatory effects of immunotherapeutics. The combination should trigger a series of innate and adaptive anti-tumor immune responses in NK cells, T cells, and macrophages [139,140]. Contradictory to conventional chemotherapy, which can cause dramatic and sudden immunosuppression in patients, another advantage of MC is that it maintains the bone marrow functions and helps maintain an immune environment suitable for immunotherapy [141,142]. As indicated in the following sections, there appears to be a great deal of evidence to support this in the context of metronomic dosing for the treatment of breast, brain, ovarian, and non-small-cell lung cancers.

#### 4.2.1. MC and Breast Cancer

The most common chemotherapeutic drugs that are used in breast cancer metronomic trials are cyclophosphamide (CTX), methotrexate (MTX), and capecitabine (CAPE). A Phase II trial in patients with metastatic breast cancer assessed metronomic low-dose capecitabine and oral CTX, and the results suggested a significant reduction in the median VEGF level in the serum [143,144]. In another Phase II trial, Bottini et al. assessed the potential anti-angiogenic effect of metronomic CTX in elderly breast cancer patients. The results of letrozole plus oral metronomic CTX therapy demonstrated a significant reduction in VEGF-A levels in the blood compared to patients treated with just letrozole. The OS rate in the letrozole/CTX treatment was higher (87.7%) than the letrozole-treated group (71.9%) [145]. In a Phase III clinical trial, Patrizia et al. demonstrated the benefit of MC with CTX and MTX. The viability of circulating endothelial cells, a potential indicator of angiogenesis, correlated with the PFS and OS rate [144,146]. Another Phase II study in patients with HER2-negative metastatic breast cancer evaluated the effectiveness of metronomic oral combination chemotherapy (CAPE (828 mg/m^2^ twice daily)) and CTX (33 mg/m^2^ twice daily, days 1–14 every 3 weeks). The overall response rate (ORR) and the median PFS were 44.4% and 12.3 months, both outperforming the expected results that would have been achieved using conventional polychemotherapy [147,148]. The benefits of MC have been attributed to the low toxicity of the milder dosing regimen and, more importantly, the vascular normalization effects [147,148,149]. It is important to note that even with the evidence supporting metronomic CTX as a single agent and in combination therapies, in these studies, the results indicated that both CD8+ and CD4+ T cells are very sensitive to the CTX dose [150,151]. This requires the dose regimen in the clinic to be carefully designed, especially when used in combination with ICIs. It will not be useful if the treatment being used to achieve TVN also suppresses beneficial immune cell functions.

#### 4.2.2. MC and Non-Small-Cell Lung Cancer (NSCLC)

The beneficial effects of TVN extend beyond breast cancer. Common chemotherapeutic drugs that are frequently being used in NSCLC metronomic trials, single or in combination, are vinorelbine, cisplatin, paclitaxel (PTX), and gemcitabine (GEM) [152]. Results of a Phase II trial showed that MC with oral vinorelbine in elderly patients with advanced NSCLC is safe, with an ORR rate and median OS of 18.6% and 9 months, respectively [152]. However, only patients with low levels of pro-angiogenic factors IL-8 and bFGF benefited significantly from the metronomic dosing of vinorelbine [152,153]. Also, most responders to metronomic vinorelbine were those that had sharp decreases in blood VEGF levels during the therapy [154,155]. This is perhaps because blood IL-8 level is not hugely affected by metronomic oral vinorelbine therapy, as demonstrated in a pre-clinical Lewis lung cancer model [156]. Katsaounis et al. investigated the therapeutic activity of oral metronomic vinorelbine (60 mg total dose, every other day) in combination with cisplatin (80 mg/m^2^) in NSCLC patients. Results showed a 1-year survival rate of 52.6% as well as stable disease in 28.6% of the patients [157]. In one study, the metronomic dosing of fractioned cisplatin and oral etoposide alone induced a significant decrease in serum VEGF, VEGF transporting cells, and Ang levels that was comparable to other groups that were on the same dose regimen with the addition of anti-VEGF mAbs. [158] Although not many clinical trials demonstrated the TVN effects of metronomic PTX and GEM in lung cancer patients, the use of both drugs is supported pre-clinically. In the murine syngeneic Lewis lung cancer model, oral metronomic GEM reduced circulating Treg cells and increased CD3^+^CD4^+^ and CD3^+^CD8^+^ T cell infiltration into the tumors compared to GEM given at the maximum tolerated dose [159]. Similarly, metronomic PTX was found to favor DC maturation, reducing microvessel density in the same tumor model [160].

#### 4.2.3. MC and Ovarian Cancer

Chemotherapy with combinations of platinum-based (such as cisplatin and carboplatin) and taxane-based (paclitaxel or docetaxel) agents is considered a first-line treatment for patients with advanced ovarian cancer (OC) [161]. However, for patients with recurrent, platinum-resistant, and platinum-refractory ovarian cancer disease, the therapeutic options are limited [162]. Various clinical trials have studied oral MC with CTX, CAPE, etoposide, and vinorelbine for these patients, some involving combining MC CTX given orally with bevacizumab [163,164]. In the Phase II study reported by Garcia et al., a 24% partial response was achieved in 70 advanced OC patients treated with a combination of bevacizumab and oral metronomic CTX, which was better than the expected response rate of bevacizumab monotherapy (17%) for advanced OC patients [163]. Unlike NSCLC, however, the plasma VEGF level could not be correlated to outcomes. In patients with high-grade serous ovarian cancer, metronomic CTX successfully induced long-term remission, which was thought to be largely attributable to the inhibition of ECs [165]. For patients with ovarian cancer, MC is most often used to prevent disease progression, and the effects are best achieved with another anti-angiogenic therapy [166]. As one example, a Phase II clinical study in patients with platinum-refractory ovarian cancer, a dual anti-angiogenic and anti-proliferative effect was achieved by combining MC and anti-VEGF antibodies with ICIs, benefits that could be attributed to the normalization of tumor blood vessels and the depletion of Treg cells [167].

#### 4.2.4. MC and Glioblastoma

When considering what is viewed as an immune-privileged site, glioblastomas (GBMs) and malignant gliomas represent a significant clinical challenge. These patients have a median survival of only 1 year and a 5-year survival of only 6.8% [168,169]. The chemotherapy drug used most often to treat brain tumors is temozolomide (TMZ) [170]. The limiting factors associated with the conventional TMZ chemotherapy are severe toxicity as well as tumor regrowth between the treatment-free intervals [170]. MC appears to provide improvements in the anti-angiogenic activity of TMZ while also reducing the drug’s toxicity. A pilot study exploring metronomic TMZ treatment (daily dose of 40 mg/m^2^) in patients with recurrent GBM reduced the emergence of chemoresistance in patients and the median OS and PFS were 11.0 and 6.0 months, respectively [171]. Another study in patients with recurrent GBM after initial TMZ/radiotherapy assessing daily TMZ at 50 mg/m^2^ showed excellent tolerability with a 6-month PFS of 57% that is slightly better than historical standard TMZ treatment outcomes [171,172].

Nevertheless, some authors have suggested that the combination of MC with other therapeutics may not provide improved treatment outcomes in the GBM patient population [173,174]. Indeed, past efforts attempting to achieve TVN or to modulate the immunosuppressive TME within GBM have been most disappointing. This may be because the site is immunologically privileged and the vasculature defining the blood–brain barrier (BBB) is unique. Further, this could be due to an abundance of Treg cells, the high expression of Ang-2, and cerebral edema [175,176,177]. GBM is infiltrative in nature, and the location of the residual tumor (after surgery) is typically unreachable for chemotherapies and therapeutic antibodies (such as targeted anti-VEGF antibodies) because of the BBB [178]. Thus, anti-VEGF mAbs and/or MC may require an innovative way to be delivered to the tumor site in order to generate enough TVN effects to enhance immunotherapies for GBM patients.

As indicated above, approaches involving the induction of TVN have been applied in many cancers like GBM and NSCLC. Further, other authors have highlighted the benefits for the treatment of patients with metastatic castration-resistant prostate cancer and hepatocellular carcinoma (HCC) [179,180]. Without a doubt, MC is an effective immunomodulatory method that can be applied widely to various cancer types. However, it can be argued that even with metronomic CTX, the drug that is perhaps most extensively investigated for MC, its optimal dose regimen and method of administration, especially when combined with immunotherapeutics, remain undefined. Therefore, chemotherapies that involve drugs which have a significant impact on the viability and functionality of key immune cells (as discussed in Section 3) may not be the best choice.

### 4.3. Nanoformulations and TVN: Defining a Platform to Augment the Activity of Immunotherapeutics

As argued here and by other investigators, it can be suggested that a key to enhancing the activity of immunotherapeutics used to treat cancer could be through the induction of TVN. However, the agents typically selected are agents that are already approved for use in a specific way. While it is easy to initiate clinical studies with these approved agents, the approved use (dose, route of administration, administration schedule) may not be best suited for combining with ICIs or for use in combination with other immunotherapies. Further, the agents used may not be most suitable to achieve vascular normalization while also promoting anti-tumor immune reactions. The systematic administration of anti-VEGF mAbs at a high dose, as one example, can cause severe cardiovascular adverse effects such as hypertension, thromboembolic disease, and myocardial ischemia [181]. These adverse effects may be complicated in the context of an individual with cancer. There is some pre-clinical evidence to suggest that TVN may be best achieved when the therapeutic agents are formulated as a component of a drug delivery system, a technology that can also mitigate toxic side effects and enhance tumor specific targeting [182].

Nanotechnologies that are better able to deliver selected agents may provide an effective approach when the goal is to achieve TVN [182,183], and nanotechnologies offer an ideal approach to define a combination product [178,179]. In the past 30 years, lipid-based nanoparticles (liposomes and lipid nanoparticles) have proven to be the most broadly approved delivery system for therapeutics [184]. The mean diameter of liposomes and other nanoparticles used intravenously is most typically around 50–150 nm [185,186]. One consequence of the size is that the liposomes and the associated drugs stay in the circulation for a long time compared to free drugs (hours vs. minutes) [187,188]. This is because normal blood vessels are organized in a manner that prevents large molecules in the blood from moving into tissues. However, as mentioned, the vascular structure within tumors is formed rapidly and poorly. These newly formed/co-opted blood vessels lack a basement membrane, and the normal tight junctions between the endothelial cells that help form the blood vessel are absent [8,13]. The gaps/fenestrations between the endothelial cells permit large particles, like liposomes, to pass into the tissue [189]. When this “leaky” blood vessel structure is in an environment that lacks a lymphatic system (a system that can remove fluid from the tissue), the extravasated material becomes trapped in the tissue. This has been referred to as the enhanced permeability and retention or EPR effect [185]. It is recognized that the regions where the tumor-associated blood vessels are leaky are very heterogeneous, and there are regions in the tumor that are “serviced” by more normal blood vessels [190,191,192]. This consequently causes the EPR effect to be heterogeneous as well [193,194]. In this context, the two core concepts of designing liposomal and polymeric nanoparticles are to (1) achieve the sustained release kinetics of the associated drugs and (2) improve the nanoparticles’ ability to be retained in the blood compartment for extended time periods [195,196,197]. These nanoformulations may provide a better way of achieving low levels of a selected drug or drugs over extended time periods in a way that may be better than MC. The nanoformulations can be designed to expose the vascular endothelial and tumor cells continuously to a low concentration of the selected agent(s) [134,198], which then provides a potential solution to one of the most common challenges associated with many metronomic-dosing chemotherapies, particularly drugs that are administered orally because they often exhibit unfavorable pharmacokinetic profiles, such as low bioavailability [199,200]. Moreover, it is continued to be believed that poor patient compliance is an issue because a lot of the benefits of MC, such as reduced harmful drug–drug interactions and increased therapeutic effects, greatly rely on the optimal dosing schedule and are sensitive to the blood concentration of one or several drugs [201]. The nanoformulation approach is thus ideally suited to define drug combination products, since such products can co-deliver more than one agent in a manner that can control the drug–drug ratio to achieve the best therapeutic effect, exposure time, and perhaps sequencing [202,203,204,205]. These advantages make nanoformulation methods ideal for strategies that are trying to alter the TME in a manner optimal for use in combination with immunotherapies.

#### A Liposomal Drug Formulation That May Be Ideal for Engendering Changes in the TME

The first time our research team recognized the potential for a liposomal formulation to alter the TME was following the completion of studies with a liposomal irinotecan (CPT-11) formulation referred to as IrC^TM^. Our team was able to demonstrate that CPT-11, when formulated into liposomes and administered intravenously, was able to engender TVN. When assessing the TVN effects, it was realized that the TME changed remarkably following treatment. For example, in Rag2M mice bearing a HT-29-derived human colorectal tumor, treatment (Q7Dx2) with IrC^TM^ resulted in decreases in tumor cell density and CD31+ endothelial cells [58,134]. These changes were associated with a 2–3-fold decrease in pro-angiogenic factor (VEGF-A, VEGF-C) expression in the tumor as well as a 4-fold upregulation of anti-angiogenic factor TIMP-1 [58]. Histopathology analysis confirmed a decrease in tumor hypoxia and an increase in tumor perfusion [58]. All effects were indicative of TVN [54]. It was expected that TVN would enhance the delivery of small-molecular-weight drugs, and it was shown that 5-fluoracil (5-FU) and doxorubicin accumulated better in the tumor of those animals previously treated with IrC^TM^ [58]. The strategy of first treating the mice with IrC^TM^ to engender TVN and subsequently administer 5-FU resulted in enhanced 5-FU levels in the tumor, significantly slowed tumor growth, and prolonged survival [200,206]. When comparing this treatment plan to the monotherapy of either IrC^TM^ or 5-FU, this sequential delivery combination method resulted in a much slower tumor growth, partially owing to the synergistic effect of the two agents but also due to the TVN effects of IrC^TM^ [134]. Therefore, our team is arguing that it is worthwhile investigating whether the treatment with an optimal CPT-11 liposomal formulation could improve the effects of immunotherapeutics, not necessarily by the improved delivery of the immunotherapeutic, but by TVN effects that change the tumor immune microenvironment by promoting immune cell infiltration [207].

It should be noted that CPT-11 is not the only drug that, when formulated in liposomes, generated a TVN effect. Liposomal doxorubicin (DOX) and vincristine have demonstrated a TVN effect [178,208]. In a study using subcutaneous and orthotopic GBM models, Verreault et al. obtained data that suggested IrC^TM^, liposomal doxorubicin (Caelyx^®^), and liposomal vincristine caused significant changes in VEGF-2 expression and CD31 expression. Magnetic resonance imaging suggested that the vascular permeability/flow (K_trans_) was reduced, indicating that the blood vessels became less leaky, and the normal blood flow in the tumor increased [203,208]. Treatment with one liposomal DOX formulation successfully inhibited the tumor ECs and reduced microvessel density after a few treatment cycles, and the effects were durable even 7 days post treatment [209]. However, continuous dosing was needed to maintain the improved tumor perfusion [209]. It could be argued that to maintain the TVN effect and to achieve benefits when combined with immunotherapies, it may be best to design the liposomal formulation to be long circulating and continuously releasing the associated drug(s). Fan et al. provided data to suggest that the combination of liposomal imatinib (20 mg/kg) and a liposomal DOX (1 mg/kg) formulation was synergistic, and the primary driving force for this synergy was the result of TVN and a reduction in tumor IFP [63]. It has also been suggested that only when theses cytotoxic drugs are formulated into nanoformulations can they synergize with anti-PD-L1 and anti-CTLA-4 antibodies [210,211]. Many underlying mechanisms had been hypothesized for beneficial combination effects. For example, in a CT-26 cancer model, the effect was thought to be mainly due to the nanoformulation’s ability to deplete Treg cells and to increase CD8+ T cell infiltration [210]. It is worthy noting that when used in the context of inducing TVN, the dose of liposomal DOX was often kept very low [63,209]. When considering combinations with immunotherapeutics, DOX may not be the best choice. This drug, particularly when used in a liposomal formulation, is toxic to macrophages, and if this effect extended to other antigen-presenting cells (APCs), then the drug would not be appropriate [212,213].

When formulating liposomal drugs to induce a TVN effect, the drug of choice is important as well as the dose. PTX was formulated in a cationic liposome and first reported to have an anti-angiogenic effect back in 2004 [209]. Following treatment (5 mg/kg) with the defined liposomal PTX formulation, the authors noted an increase in TV permeability and reduced functional microvessel density in the tumor [214,215]. It has always been thought that PTX exhibits mainly anti-angiogenic effects [216]. Nevertheless, another study suggested that PTX could help to induce TVN when delivered by a dextran-deoxycholic acid-based nanoparticle formulate when used in combination with silybin. The TVN effect was best achieved when the combination of silybin and PTX was given at a moderate dose and was released at an optimal release ratio. One design difference between the two formulations was perhaps when formulated in a dextran-deoxycholic acid-based nanoparticle; this new formulation allowed the PTX to target the cancer-associated fibroblast and subsequently alleviated the compression pressure from collagen secretion on the blood vessels [217]. Although the TVN effect was not entirely attributable to PTX, this study successfully demonstrated the potential of using PTX in a combination therapy to achieve TVN [217]. Other teams also attempted to combine selected liposomal PTX formulations with other anti-angiogenic agents in order to achieve improved TVN effects, better then what could be expected from the individual agents [218].

Liposomes have also been used to deliver novel agents that can then be combined in various ways to modulate the immune system through a TVN effect [219,220]. Cai et al. formulated zoledronic acid into polyethylene glycol (PEG)-modified cationic liposomes. The formulation caused significant decreases in tumor microvessel density, repressed tumor hypoxia, and worked at least additively when combined with free cisplatin [220]. When using mice-bearing syngeneic CT-26 colorectal tumors, Luput et al. demonstrated that the sequential delivery of simvastatin and 5-FU by liposomes worked well together, arguing that the simvastatin induced TVN and sensitized the tumor to 5-FU. The treatment resulted in a significant decrease (>80%) in a panel of pro-angiogenic factors including IL-1β, IL-6, bFGF, and VEGF that are indicative of the vascular normalization process [221]. A different research team developed a complex lipid-based drug delivery system encapsulating topotecan (as an approved anti-cancer drug), indocyanine green (used as a sensitizing agent), and with erlotinib (epidermal growth factor receptor inhibitor) associated on the particle surface with electronic interaction. The TVN effect that the authors observed was prolonged, and this was thought to be due to a combination of the topotecan-mediated inhibition of HIF-1α and erlotinib-mediated normalizing TV [222].

It is suggested here that the potential of some drugs, such as CPT-11 and zoledronic acid, to modulate the TME may have been overlooked when considering the actions of the agents given “free” rather than as a nanoformulation of the drug. Moreover, the above evidence with PTX also illustrated that the design of the drug delivery system is critically important because the therapeutic agent needs to be released in a way that enhances the killing of abnormal microblood vessels rather than inhibiting blood vessel formation in general. As suggested above, the TVN effect is mainly due to changes in the drug’s PK profile that mimic metronomic dosing. Others suggest that the liposome-encapsulated/associated drug(s) are better when internalized into endosomes and that processing and distribution is different compared to the free drug [223,224,225]. Therefore, further mechanistic studies are crucial to help explore liposomal drugs as an immunomodulatory adjuvant to immunotherapy. Nevertheless, we believe the aggregate of data compels an emphasis on combination therapy, especially the co-delivering of drugs by liposomes to achieve immunomodulation of the TME.

## 5. Discussion and Comment on Future Directions

With a lot of progress made in the past decade in nanomedicine and with the successful development of the mRNA lipid nanoparticle (LNP) technologies, cancer therapies are entering an era where therapeutic nucleic acids might provide new methods to modulate the TME through changing the tumor vasculature towards one that favours immune cell migration into the tumor and augmentation of immunotherapeutics. For example, the siRNA-mediated knockdown of VEGF signalling has gained much attention for regulating the TME. Sakurai et al. demonstrated that the siRNA-LNP inhibition of VEGFR-2 led to vascular normalization in a hypovascular cancer model [226]. The application of RNA interference (RNAi) technology to achieve TVN has been evaluated through the inhibition of VEGF. Tabernero et al. introduced the first-in-class LNP-formulated RNAi-mediated gene silencing of VEGF in patients with liver cancer [227]. Xing et al. demonstrated that VEGF suppression by RNAi led to apoptosis induction, angiogenesis reduction, and radiosensitivity enhancement in a cervical cancer xenograft mouse model [228]. Perhaps the collective efforts of silencing the VEGF signalling pathway and ICIs could re-boost T cell functions and enhance the therapeutic outcomes of ICIs. The efficacy of ICIs is dependent on many factors, but it is strongly believed that the efficacy of ICIs relies on the reactivation and clonal proliferation of T cells in the TME [229].

Various LNP formulations have been shown to improve the anti-tumor response rate with ICIs through TVN. It can be suggested that these are very early days in which oncologists are trying to establish how best to significantly enhance the effects of immunotherapeutics. Some clinical studies designed to explore the combinations of known agents approved for use in the treatment of cancer with immunotherapeutics are ongoing. Other investigators are exploring novel therapeutic agents or existing agents approved for non-oncology indications. Nevertheless, how long will it take to define combinations that change the standard of care for patients? It could be argued that this approach has been fast tracked simply because there are many potential combinations that can be selected from a large menu of approved agents. Alternatively, the immune suppressive TME along with the lack of recognizable neoantigens may be the limiting factors. Finally, it can be further suggested that immunotherapies may only be suitable for selected tumor types (e.g., lung, melanoma) [230,231].

However, as one example, it should be noted that in patients with bladder cancer that have become metastatic and insensitive to cisplatin, most all are treated with ICIs. The response rate in this patient population is limited to about 20%, and the 1-year OS rate is improved to about 20%, and this has been referred to as a “significant” advance [232]. For those working in the pre-clinical space, like our team that has authored this paper, there are significant challenges in part due to the fact that pre-clinical efficacy studies need to be completed in immune-competent mice, mice bearing established syngeneic tumors. Such tumor models were the standard defined by the US NCI between 1960 and 1980 and were useful in the identification of many of the anti-cancer drugs that are used routinely today. There was a change in models when immune-incompetent mice were created, mice that allowed the growth of human tumor cell lines and patient-derived tumors. Obviously, these models are not suitable to assess the potential of combinations with immunotherapeutics. To address this, investigators are developing humanized mice that have human immune cells and can also grow human tumors/tumor cell lines. These, however, also have limitations, cost being one, which limit the ability of academics to access them.

Our team is trying to take advantage of small-molecular-weight compounds that can enhance the activity of immunotherapy. With this goal and the background provided above, our interests lie in the use of compounds that can engender TVN and ICD. While the TVN effect appears, based on the current literature, to provide benefits, it may be also critical to ensure maximum exposure to tumor factors that may lead to tumor antigen recognition by APCs. In our opinion, this could be through the use of agents that promote TVN as well as mediate tumor cell ICD.

This approach may prove to benefit the activity of ICIs as well as the activity of CAR-T cells. However, safety considerations need to be made. For example, CAR-T cell targeting of CD19-positive cancer cells are clinically successful for the treatment of hematologic cancers (e.g., acute and chronic B cell leukemia) [233]. However, this approach is proving to have little value for the treatment of solid tumours due to the physical TME barriers that are not present in hematologic cancers [233,234]. As indicated in this review, certain cardiovascular drugs may alleviate the effects of a hostile TME and reduce the presence of certain suppressive immune cells [97,234]. However, the value of this combination approach has yet to be established clinically. The cardiovascular drugs identified above may be most suitable for use in combination with CAR-T cells. Approaches that involve chemotherapies and radiation will suppress white blood cells and the functionality associated with T cells and neutrophil-assisted T cell activations [235,236]. However, the use of anti-angiogenic antibodies (e.g., bevacizumab and ranibizumab) may create multi-site bleeding disorders, a complication that may contribute to CAR-T-cell-induced systematic cytokine release syndrome [237,238].

We have highlighted concepts that may modulate the TME by mediating changes in tumor-associated vasculature. Tumor vascular normalization effects have immunomodulatory effects that should augment the activity of immunotherapies. Many studies that have been disclosed at this time (2023) suggest that there are approaches that should be safe, but this needs to be determined in the clinic. The trend of inducing vascular normalization has shifted from using anti-VEGF antibodies to repurposing cardiovascular drugs to, in our view, the use of nanomedicines that exert anti-angiogenic effects. At one level, these approaches may seem old school, as many will suggest that future medicines will be genetic medicines designed to increase, decrease, or silence a selected gene(s). Regardless, it is hoped that this review will provide references and inspiration for more cancer and nanomedicine researchers to appreciate the importance of tumor-associated blood vessels to augment therapeutic outcomes when used in combination with other treatments, in particular immunotherapeutics.

## Figures and Tables

**Table 1:** Summary of US Food and Drug Administration (FDA) approved immunotherapies with their targets, underlying mechanisms, and approved tumor type indications [1,3,4,5].

	Generic Name	Target *	Mechanism	Approved for **
Immune checkpoint inhibitors	Ipilimumab	CTLA-4	Inhibit CTLA-4 and increase T cell activation	Melanoma, RCC, MCC, HCC, Metastatic NSCLC
	Cemiplimab	PD-1	Inhibit PD-1 and increase T cell activation	Squamous Cell Carcinoma, NSCLC
	Nivolumab	PD-1	Inhibit PD-1 and increase T cell activation	Melanoma, Lung cancer, NSCLC, RCC, Hodgkin’s Lymphoma, Head and Neck Cancer, MUC, MCC, HCC NSCLC, Esophageal Carcinoma, Gastric Cancer
	Pembrolizumab	PD-1	Inhibit PD-1 and increase T cell activation	Advanced Melanoma, Advanced NSCLC, Head and Neck Cancer, Hodgkin’s Lymphoma, MUC, Gastric Cancer, Cervical Cancer, HCC, Merkel Cell Carcinoma, RCC, Endometrial Cancer, Squamous Cell Carcinoma, HCC, Breast Cancer
	Atezolizumab	PD-L1	Inhibit PD-L1 and increase T cell activation	NSCLC, Small Cell Lung Cancer, HCC, Melanoma
	Avelumab	PD-L1	Inhibit PD-L1 and increase T cell activation	Merkel Cell Carcinoma, Urothelial Carcinoma, RCC
	Durvalumab	PD-L1	Inhibit PD-L1 and increase T cell activation	NSCLC, Small Cell Lung Cancer, Biliary Tract Tumor
Cytokine-based therapies	Aldesleukin	IL-2 receptor	Increase T cell activation	Metastatic Melanoma and Metastatic Renal Cell Carcinoma.
	Interferon alpha-2b	Type I IFN receptors	Activate type 1 IFN receptors and JAK/STAT pathway	Leukemia, Follicular Lymphoma, Malignant Melanoma, AIDs-related Kaposi’s Sarcoma
CAR-T cell therapies	Tisagenlecleucel	CD19	T cell activation, expansion and elimination of target cells	ALL, NHL
	Axicabtagene ciloleucel	CD19	T cell activation, expansion and elimination of target cells	NHL, Follicular Lymphoma
	Brexucabtagene autoleucel	CD19	T cell activation, expansion and elimination of target cells	Mantle Cell Lymphoma, ALL
	Lisocabtagene maraleucel	CD19	T cell activation, expansion and elimination of target cells	NHL
	Idecabtagene vicleucel	BCMA	T cell activation, expansion and elimination of target cells	Multiple Myeloma
	Ciltacabtagene autoleucel	BCMA	T cell activation, expansion and elimination of target cells	Multiple Myeloma
Vaccine	Sipuleucel-T	Prostatic acid phosphatase	Induce immune activation towards prostate cancer cells	Metastatic Prostate Cancer

* Target abbreviations: PD-1/PD-L1: programmed cell death protein-1/programmed cell death ligand-1, CTLA-4: cytotoxic T-lymphocyte-associated protein 4, IL-2: interleukin 2, CD19: cluster of differentiation 19 molecule, BCMA: B-cell maturation antigen, IFN: interferon. ** Disease abbreviation: CDC: complement-dependent cytotoxicity, ADCC: antibody-dependant cellular cytotoxicity, ADCP: antibody-dependent cellular phagocytosis, CLL: chronic lymphocytic leukemia, NHL: non-Hodgkin’s lymphoma, NSCLC: non-small-cell lung cancer, MBC: metastatic breast cancer, MSC: metastatic stomach cancer. HCC: hepatocellular carcinoma, MCC: metastatic colorectal cancer, MUC: metastatic urothelial carcinoma, RCC: renal cell carcinoma, ALL: acute lymphoblastic leukemia.

**Table 2:** Summary of the major classes of cardiovascular drugs that are being considered for repurposing and use in combination with immunotherapies to treat cancers including angiotensin receptor blockers (ARBs), beta-blockers, cardiac glycosides (CGs), and cyclooxygenase (COX) inhibitors. While the ARBs, beta-blockers, and COX inhibitors exert TVN effects, the cardiac glycosides appear to enhance immunotherapy outcomes by the induction of ICD.

Drug Class	Drug Names	Target	Affected Cancer Types	Beneficial Effect on Immunotherapy	References
ARBs	Telmisartan, Losartan, Candesartan	Angiotensin type 1 receptor	Breast, pancreatic ductal adenocarcinoma, bladder	- ↓ VEGF and pro-inflammatory factors - modulate vasculature of the TME - modulate NF-κB and HIF-1 expression	[97,98,99,100,101]
Beta-blockers	Propranolol, Metoprolol	β1, β2, β3 receptor	Melanoma, breast, ovarian, colorectal	- mediate stress response - improve T cell anti-tumor response - ↓ accumulation of immunosuppressive cells	[102,103,104]
Cyclooxygenase Inhibitors	Aspirin	Cyclooxygenase enzyme	Colorectal	- ↓ VEGF - ↓ accumulation of immunosuppressive cells - ↓ IL-6 and IL-10 - ↑ IL-12 and IFNγ	[105,106,107]
Cardiac Glycosides	Oleandrin, Scillaren A, Proscillaridin, Lanatoside C, Digitoxigenin	Sodium-potassium ATPase pump	Breast	- exert immunogenic cell death - modulate FGF-2 and NF-κB - ↓ IL-10 - ↑ IL-2 and IFNγ	[108,109,110]

↑ = increase, ↓ = decrease.

## Abbreviations

ICIs—immune checkpoint inhibitors; CAR-T cells—chimeric antigen receptor T cells; TME—tumor microenvironment; TV—tumor vasculature; PFS—progression free survival; OS—overall survival; TVN—tumor vascular normalization; CTLA-4—cytotoxic T-lymphocyte-associated protein 4; VEGF—vascular endothelial growth factor; bFGF—basic fibroblast growth factor; FGF—fibroblast growth factor; TGF-β—transforming growth factor β; IL—interleukin; HIF—hypoxia inducible factor; NF-κB—nuclear factor kappa-light-chain-enhancer of activated B cells; MCP1—monocyte chemoattractant protein-1; TAM/M2s—tumor-associated macrophages/type 2 macrophages; DCs—dendritic cells; CTL—cytotoxic T cell; CD—cluster of differentiation; PDGF—platelet-derived growth factor; EGF—epidermal growth factor; VEGFR—vascular endothelial growth factor receptor; IFN-γ—interferon-γ; CCL—chemokine (C-C motif) ligand; IFP—interstitial fluid pressure; M1—pro-inflammatory type 1 macrophage; NK cell—natural killer cell; STING—stimulator of interferon gene; TNBC—triple negative breast cancer; Ang 2—angiopoietin 2; MDSCs—myeloid-derived suppressor cells; ARB—angiotensin receptor blocker; CGs—cardiac glycosides; COX—cyclooxygenase; RAAS—renin angiotensin aldosterone system; Angt II—angiotensin II; mAb—monoclonal antibody; Treg—regulatory T cell; AT1R—Angt II type 1 receptor; ACE/ACE I—angiotensin-converting enzyme/angiotensin-converting enzyme inhibitor; ECM—extracellular matrix; CAF—cancer-associated fibroblast; β-ARs—beta-adrenoceptors; PGE-2—prostaglandin E2; ICD—immunogenic cell death; MC—metronomic chemotherapy; EC—endothelial cell; OXP—oxaliplatin; DOX—doxorubicin; CTX—cyclophosphamide; DAMP—damage-associated molecular pattern; HMGB-1—high-mobility group box 1; CRT—calreticulin; CTX—cyclophosphamide; MTX—methotrexate; CAPE—capecitabine; NSCLC—non-small-cell lung cancer; PTX—paclitaxel; GEM—gemcitabine; OC—ovarian cancer; GBM—glioblastoma; TMZ—temozolomide; HCC—hepatocellular carcinoma; CPT-11—irinotecan; LNP—lipid nanoparticle; PEG—polyethylene glycol; EPR—enhanced permeability and retention; BBB—blood–brain barrier; RNAi—RNA interference; APC—antigen-presenting cell.

## References

[1] Esfahani K., Roudaia L., Buhlaiga N., Del Rincon S.V., Papneja N., Miller W.H. (2020). A Review of Cancer Immunotherapy: From the Past, to the Present, to the Future. Curr. Oncol..

[2] Akkın S., Varan G., Bilensoy E. (2021). A Review on Cancer Immunotherapy and Applications of Nanotechnology to Chemoimmunotherapy of Different Cancers. Molecules.

[3] Naimi A., Mohammed R.N., Raji A., Chupradit S., Yumashev A.V., Suksatan W., Shalaby M.N., Thangavelu L., Kamrava S., Shomali N. (2022). Tumor immunotherapies by immune checkpoint inhibitors (ICIs); the pros and cons. Cell Commun. Signal..

[4] Twomey J.D., Zhang B. (2021). Cancer Immunotherapy Update: FDA-Approved Checkpoint Inhibitors and Companion Diagnostics. AAPS J..

[5] Liu C., Yang M., Zhang D., Chen M., Zhu D. (2022). Clinical cancer immunotherapy: Current progress and prospects. Front. Immunol..

[6] Gonzalez H., Hagerling C., Werb Z. (2018). Roles of the immune system in cancer: From tumor initiation to metastatic progression. Genes Dev..

[7] Baghban R., Roshangar L., Jahanban-Esfahlan R., Seidi K., Ebrahimi-Kalan A., Jaymand M., Kolahian S., Javaheri T., Zare P. (2020). Tumor microenvironment complexity and therapeutic implications at a glance. Cell Commun. Signal..

[8] Jain R.K. (2005). Normalization of Tumor Vasculature: An Emerging Concept in Antiangiogenic Therapy. Science.

[9] Li S., Zhang Q., Hong Y. (2020). Tumor Vessel Normalization: A Window to Enhancing Cancer Immunotherapy. Technol. Cancer Res. Treat..

[10] Hanahan D. (2022). Hallmarks of Cancer: New Dimensions. Cancer Discov..

[11] Hall A.P. (2005). The role of angiogenesis in cancer. Comp. Clin. Pathol..

[12] Eichhorn M.E., Kleespies A., Angele M.K., Jauch K.-W., Bruns C.J. (2007). Angiogenesis in cancer: Molecular mechanisms, clinical impact. Langenbecks Arch. Surg..

[13] Lugano R., Ramachandran M., Dimberg A. (2020). Tumor angiogenesis: Causes, consequences, challenges and opportunities. Cell. Mol. Life Sci..

[14] Fu L.-Q., Du W.-L., Cai M.-H., Yao J.-Y., Zhao Y.-Y., Mou X.-Z. (2020). The roles of tumor-associated macrophages in tumor angiogenesis and metastasis. Cell. Immunol..

[15] Stockmann C., Schadendorf D., Klose R., Helfrich I. (2014). The Impact of the Immune System on Tumor: Angiogenesis and Vascular Remodeling. Front. Oncol..

[16] Batlle E., Massagué J. (2019). Transforming Growth Factor-β Signaling in Immunity and Cancer. Immunity.

[17] Sun W., Wei F.-Q., Li W.-J., Wei J.-W., Zhong H., Wen Y.-H., Lei W.-B., Chen L., Li H., Lin H.-Q. (2017). A positive-feedback loop between tumour infiltrating activated Treg cells and type 2-skewed macrophages is essential for progression of laryngeal squamous cell carcinoma. Br. J. Cancer.

[18] Khouzam R.A., Brodaczewska K., Filipiak A., Zeinelabdin N.A., Buart S., Szczylik C., Kieda C., Chouaib S. (2021). Tumor Hypoxia Regulates Immune Escape/Invasion: Influence on Angiogenesis and Potential Impact of Hypoxic Biomarkers on Cancer Therapies. Front. Immunol..

[19] Bhandari V., Hoey C., Liu L.Y., Lalonde E., Ray J., Livingstone J., Lesurf R., Shiah Y.-J., Vujcic T., Huang X. (2019). Molecular landmarks of tumor hypoxia across cancer types. Nat. Genet..

[20] Muz B., de la Puente P., Azab F., Azab A.K. (2015). The role of hypoxia in cancer progression, angiogenesis, metastasis, and resistance to therapy. Hypoxia.

[21] Yang G., Shi R., Zhang Q. (2021). Hypoxia and Oxygen-Sensing Signaling in Gene Regulation and Cancer Progression. Int. J. Mol. Sci..

[22] Prabhakar N.R., Semenza G.L. (2015). Oxygen Sensing and Homeostasis. Physiology.

[23] Korbecki J., Simińska D., Gąssowska-Dobrowolska M., Listos J., Gutowska I., Chlubek D., Baranowska-Bosiacka I. (2021). Chronic and Cycling Hypoxia: Drivers of Cancer Chronic Inflammation through HIF-1 and NF-κB Activation: A Review of the Molecular Mechanisms. Int. J. Mol. Sci..

[24] Nejad A.E., Najafgholian S., Rostami A., Sistani A., Shojaeifar S., Esparvarinha M., Nedaeinia R., Javanmard S.H., Taherian M., Ahmadlou M. (2021). The role of hypoxia in the tumor microenvironment and development of cancer stem cell: A novel approach to developing treatment. Cancer Cell Int..

[25] Wadsworth B.J., Lee C.-M., Bennewith K.L. (2022). Transiently hypoxic tumour cell turnover and radiation sensitivity in human tumour xenografts. Br. J. Cancer.

[26] Chaudary N., Hill R.P. (2009). Increased expression of metastasis-related genes in hypoxic cells sorted from cervical and lymph nodal xenograft tumors. Lab. Investig..

[27] Kierans S.J., Taylor C.T. (2020). Regulation of glycolysis by the hypoxia-inducible factor (HIF): Implications for cellular physiology. J. Physiol..

[28] Riemann A., Ihling A., Thomas J., Schneider B., Thews O., Gekle M. (2015). Acidic environment activates inflammatory programs in fibroblasts via a cAMP–MAPK pathway. Biochim. Biophys. Acta—Mol. Cell Res..

[29] Barnabei L., Laplantine E., Mbongo W., Rieux-Laucat F., Weil R. (2021). NF-κB: At the Borders of Autoimmunity and Inflammation. Front. Immunol..

[30] Nishida N., Yano H., Nishida T., Kamura T., Kojiro M. (2006). Angiogenesis in cancer. Vasc. Health Risk Manag..

[31] Cao L., Huang T., Chen X., Li W., Yang X., Zhang W., Li M., Gao R. (2021). Uncovering the interplay between pH receptors and immune cells: Potential drug targets (Review). Oncol. Rep..

[32] Díaz F.E., Dantas E., Geffner J. (2018). Unravelling the Interplay between Extracellular Acidosis and Immune Cells. Mediat. Inflamm..

[33] Rébé C., Ghiringhelli F. (2020). Interleukin-1β and Cancer. Cancers.

[34] Fischer K., Hoffmann P., Voelkl S., Meidenbauer N., Ammer J., Edinger M., Gottfried E., Schwarz S., Rothe G., Hoves S. (2007). Inhibitory effect of tumor cell–derived lactic acid on human T cells. Blood.

[35] Calcinotto A., Filipazzi P., Grioni M., Iero M., De Milito A., Ricupito A., Cova A., Canese R., Jachetti E., Rossetti M. (2012). Modulation of Microenvironment Acidity Reverses Anergy in Human and Murine Tumor-Infiltrating T Lymphocytes. Cancer Res..

[36] Tu V.Y., Ayari A., O’connor R.S. (2021). Beyond the Lactate Paradox: How Lactate and Acidity Impact T Cell Therapies against Cancer. Antibodies.

[37] Bannoud N., Dalotto-Moreno T., Kindgard L., García P.A., Blidner A.G., Mariño K.V., Rabinovich G.A., Croci D.O. (2021). Hypoxia Supports Differentiation of Terminally Exhausted CD8 T Cells. Front. Immunol..

[38] Dang E.V., Barbi J., Yang H.-Y., Jinasena D., Yu H., Zheng Y., Bordman Z., Fu J., Kim Y., Yen H.-R. (2011). Control of TH17/Treg Balance by Hypoxia-Inducible Factor 1. Cell.

[39] Zahra F.T., Sajib S., Mikelis C.M. (2021). Role of bFGF in Acquired Resistance upon Anti-VEGF Therapy in Cancer. Cancers.

[40] Stavri G.T., Zachary I.C., Baskerville P.A., Martin J.F., Erusalimsky J.D. (1995). Basic Fibroblast Growth Factor Upregulates the Expression of Vascular Endothelial Growth Factor in Vascular Smooth Muscle Cells. Circulation.

[41] Hosaka K., Yang Y., Seki T., Du Q., Jing X., He X., Wu J., Zhang Y., Morikawa H., Nakamura M. (2020). Therapeutic paradigm of dual targeting VEGF and PDGF for effectively treating FGF-2 off-target tumors. Nat. Commun..

[42] Hellberg C., Östman A., Heldin C.-H. (2010). PDGF and Vessel Maturation. Angiogenesis Inhib..

[43] Mashreghi M., Azarpara H., Bazaz M.R., Jafari A., Masoudifar A., Mirzaei H., Jaafari M.R. (2018). Angiogenesis biomarkers and their targeting ligands as potential targets for tumor angiogenesis. J. Cell. Physiol..

[44] Akwii R.G., Sajib M.S., Zahra F.T., Mikelis C.M. (2019). Role of Angiopoietin-2 in Vascular Physiology and Pathophysiology. Cells.

[45] Leong A., Kim M. (2020). The Angiopoietin-2 and TIE Pathway as a Therapeutic Target for Enhancing Antiangiogenic Therapy and Immunotherapy in Patients with Advanced Cancer. Int. J. Mol. Sci..

[46] Chanmee T., Ontong P., Konno K., Itano N. (2014). Tumor-Associated Macrophages as Major Players in the Tumor Microenvironment. Cancers.

[47] Lee W.S., Yang H., Chon H.J., Kim C. (2020). Combination of anti-angiogenic therapy and immune checkpoint blockade normalizes vascular-immune crosstalk to potentiate cancer immunity. Exp. Mol. Med..

[48] DU H., Shi H., Chen D., Zhou Y., Che G. (2015). Cross-talk between endothelial and tumor cells via basic fibroblast growth factor and vascular endothelial growth factor signaling promotes lung cancer growth and angiogenesis. Oncol. Lett..

[49] Im J.H., Buzzelli J.N., Jones K., Franchini F., Gordon-Weeks A., Markelc B., Chen J., Kim J., Cao Y., Muschel R.J. (2020). FGF2 alters macrophage polarization, tumour immunity and growth and can be targeted during radiotherapy. Nat. Commun..

[50] Cao Y., Cao R., Hedlund E.-M. (2008). R Regulation of tumor angiogenesis and metastasis by FGF and PDGF signaling pathways. J. Mol. Med..

[51] Chen C.-F., Feng X., Liao H.-Y., Jin W.-J., Zhang J., Wang Y., Gong L.-L., Liu J.-J., Yuan X.-H., Zhao B.-B. (2014). Regulation of T cell proliferation by JMJD6 and PDGF-BB during chronic hepatitis B infection. Sci. Rep..

[52] Daynes R.A., Dowell T., Araneo B.A. (1991). Platelet-derived growth factor is a potent biologic response modifier of T cells. J. Exp. Med..

[53] Van Steensel L., Paridaens D., Dingjan G.M., van Daele P.L.A., van Hagen P.M., Kuijpers R.W.A.M., Bosch W.A.v.D., Drexhage H.A., Hooijkaas H., Dik W.A. (2010). Platelet-Derived Growth Factor-BB: A Stimulus for Cytokine Production by Orbital Fibroblasts in Graves’ Ophthalmopathy. Investig. Opthalmol. Vis. Sci..

[54] Wang M., Wei J., Shang F., Zang K., Ji T. (2019). Platelet-derived growth factor B attenuates lethal sepsis through inhibition of inflammatory responses. Int. Immunopharmacol..

[55] Carmeliet P., Jain R.K. (2011). Principles and mechanisms of vessel normalization for cancer and other angiogenic diseases. Nat. Rev. Drug Discov..

[56] Lopes-Coelho F., Martins F., Pereira S.A., Serpa J. (2021). Anti-Angiogenic Therapy: Current Challenges and Future Perspectives. Int. J. Mol. Sci..

[57] Magnussen A.L., Mills I.G. (2021). Vascular normalisation as the stepping stone into tumour microenvironment transformation. Br. J. Cancer.

[58] Baker J.H., Lam J., Kyle A.H., Sy J., Oliver T., Co S.J., Dragowska W.H., Ramsay E., Anantha M., Ruth T.J. (2008). Irinophore C, a Novel Nanoformulation of Irinotecan, Alters Tumor Vascular Function and Enhances the Distribution of 5-Fluorouracil and Doxorubicin. Clin. Cancer Res..

[59] Xu Z., Guo C., Ye Q., Shi Y., Sun Y., Zhang J., Huang J., Huang Y., Zeng C., Zhang X. (2021). Endothelial deletion of SHP2 suppresses tumor angiogenesis and promotes vascular normalization. Nat. Commun..

[60] Tang D., Zhang S., Shi X., Wu J., Yin G., Tan X., Liu F., Wu X., Du X. (2019). Combination of Astragali Polysaccharide and Curcumin Improves the Morphological Structure of Tumor Vessels and Induces Tumor Vascular Normalization to Inhibit the Growth of Hepatocellular Carcinoma. Integr. Cancer Ther..

[61] Navarro R., Compte M., Álvarez-Vallina L., Sanz L. (2016). Immune Regulation by Pericytes: Modulating Innate and Adaptive Immunity. Front. Immunol..

[62] Kaushik D.K., Bhattacharya A., Lozinski B.M., Yong V.W. (2021). Pericytes as mediators of infiltration of macrophages in multiple sclerosis. J. Neuroinflamm..

[63] Fan Y., Du W., He B., Fu F., Yuan L., Wu H., Dai W., Zhang H., Wang X., Wang J. (2013). The reduction of tumor interstitial fluid pressure by liposomal imatinib and its effect on combination therapy with liposomal doxorubicin. Biomaterials.

[64] Yang T., Xiao H., Liu X., Wang Z., Zhang Q., Wei N., Guo X. (2021). Vascular Normalization: A New Window Opened for Cancer Therapies. Front. Oncol..

[65] Goel S., Wong A.H.-K., Jain R.K. (2012). Vascular Normalization as a Therapeutic Strategy for Malignant and Nonmalignant Disease. Cold Spring Harb. Perspect. Med..

[66] Bonaventura P., Shekarian T., Alcazer V., Valladeau-Guilemond J., Valsesia-Wittmann S., Amigorena S., Caux C., Depil S. (2019). Cold Tumors: A Therapeutic Challenge for Immunotherapy. Front. Immunol..

[67] Liu Y.-T., Sun Z.-J. (2021). Turning cold tumors into hot tumors by improving T-cell infiltration. Theranostics.

[68] Chelvanambi M., Fecek R.J., Taylor J.L., Storkus W.J. (2021). STING agonist-based treatment promotes vascular normalization and tertiary lymphoid structure formation in the therapeutic melanoma microenvironment. J. Immunother. Cancer.

[69] Zhang N., Yin R., Zhou P., Liu X., Fan P., Qian L., Dong L., Zhang C., Zheng X., Deng S. (2021). DLL1 orchestrates CD8^+^T cells to induce long-term vascular normalization and tumor regression. Proc. Natl. Acad. Sci. USA.

[70] Park S., Oh J.H., Park D.J., Zhang H., Noh M., Kim Y., Kim Y.-S., Kim H., Kim Y.-M., Ha S.-J. (2021). CU06-1004-Induced Vascular Normalization Improves Immunotherapy by Modulating Tumor Microenvironment via Cytotoxic T Cells. Front. Immunol..

[71] Shigeta K., Datta M., Hato T., Kitahara S., Chen I.X., Matsui A., Kikuchi H., Mamessier E., Aoki S., Ramjiawan R.R. (2020). Dual Programmed Death Receptor-1 and Vascular Endothelial Growth Factor Receptor-2 Blockade Promotes Vascular Normalization and Enhances Antitumor Immune Responses in Hepatocellular Carcinoma. Hepatology.

[72] Principe D.R., Chiec L., Mohindra N.A., Munshi H.G. (2021). Regulatory T-Cells as an Emerging Barrier to Immune Checkpoint Inhibition in Lung Cancer. Front. Oncol..

[73] Kamada T., Togashi Y., Tay C., Ha D., Sasaki A., Nakamura Y., Sato E., Fukuoka S., Tada Y., Tanaka A. (2019). PD-1^+^ regulatory T cells amplified by PD-1 blockade promote hyperprogression of cancer. Proc. Natl. Acad. Sci. USA.

[74] Sharma A., Subudhi S.K., Blando J., Scutti J., Vence L., Wargo J., Allison J.P., Ribas A., Sharma P. (2019). Anti-CTLA-4 Immunotherapy Does Not Deplete FOXP3+ Regulatory T Cells (Tregs) in Human Cancers. Clin. Cancer Res..

[75] Facciabene A., Coukos G. (2012). Abstract 308: Tumor hypoxia promotes tolerance and angiogenesis via CCL28 and Treg cells. Cancer Res..

[76] Riabov V., Gudima A., Wang N., Mickley A., Orekhov A., Kzhyshkowska J. (2014). Role of tumor associated macrophages in tumor angiogenesis and lymphangiogenesis. Front. Physiol..

[77] Mirando A.C., Patil A., Rafie C.I., Christmas B.J., Pandey N.B., Stearns V., Jaffee E.M., Torres E.T.R., Popel A.S. (2020). Regulation of the tumor immune microenvironment and vascular normalization in TNBC murine models by a novel peptide. Oncoimmunology.

[78] Zhou J., Li Y., Shi X., Hao S., Zhang F., Guo Z., Gao Y., Guo H., Liu L. (2021). Oridonin inhibits tumor angiogenesis and induces vessel normalization in experimental colon cancer. J. Cancer.

[79] Kloepper J., Riedemann L., Amoozgar Z., Seano G., Susek K., Yu V., Dalvie N., Amelung R.L., Datta M., Song J.W. (2016). Ang-2/VEGF bispecific antibody reprograms macrophages and resident microglia to anti-tumor phenotype and prolongs glioblastoma survival. Proc. Natl. Acad. Sci. USA.

[80] Lee C., Jeong H., Bae Y., Shin K., Kang S., Kim H., Oh J., Bae H. (2019). Targeting of M2-like tumor-associated macrophages with a melittin-based pro-apoptotic peptide. J. Immunother. Cancer.

[81] Rolny C., Mazzone M., Tugues S., Laoui D., Johansson I., Coulon C., Squadrito M.L., Segura I., Li X., Knevels E. (2011). HRG Inhibits Tumor Growth and Metastasis by Inducing Macrophage Polarization and Vessel Normalization through Downregulation of PlGF. Cancer Cell.

[82] Bastien J.-P., Minguy A., Dave V., Roy D.C. (2019). Cellular therapy approaches harnessing the power of the immune system for personalized cancer treatment. Semin. Immunol..

[83] Gabrilovich D., Ishida T., Oyama T., Ran S., Kravtsov V., Nadaf S., Carbone D.P. (1998). Vascular Endothelial Growth Factor Inhibits the Development of Dendritic Cells and Dramatically Affects the Differentiation of Multiple Hematopoietic Lineages In Vivo. Blood.

[84] Peng Q., Qiu X., Zhang Z., Zhang S., Zhang Y., Liang Y., Guo J., Peng H., Chen M., Fu Y.-X. (2020). PD-L1 on dendritic cells attenuates T cell activation and regulates response to immune checkpoint blockade. Nat. Commun..

[85] Shitara K., Nishikawa H. (2016). Regulatory T cells: A potential target in cancer immunotherapy. Ann. N. Y. Acad. Sci..

[86] Boucher Y., Kumar A.S., Posada J.M., Gjini E., Pfaff K., Lipschitz M., Lako A., Duda D.G., Rodig S.J., Hodi F.S. (2021). Bevacizumab improves tumor infiltration of mature dendritic cells and effector T-cells in triple-negative breast cancer patients. npj Precis. Oncol..

[87] Wooster A.L., Girgis L.H., Brazeale H., Anderson T.S., Wood L.M., Lowe D.B. (2021). Dendritic cell vaccine therapy for colorectal cancer. Pharmacol. Res..

[88] Fu C., Jiang A. (2018). Dendritic Cells and CD8 T Cell Immunity in Tumor Microenvironment. Front. Immunol..

[89] Foy K.C., Miller M.J., Moldovan N., Carson W.E., Kaumaya P.T.P. (2012). Combined vaccination with HER-2 peptide followed by therapy with VEGF peptide mimics exerts effective anti-tumor and anti-angiogenic effects in vitro and in vivo. Oncoimmunology.

[90] Manning E.A., Ullman J.G., Leatherman J.M., Asquith J.M., Hansen T.R., Armstrong T.D., Hicklin D.J., Jaffee E.M., Emens L.A. (2007). A Vascular Endothelial Growth Factor Receptor-2 Inhibitor Enhances Antitumor Immunity through an Immune-Based Mechanism. Clin. Cancer Res..

[91] Renner D.N., Malo C.S., Jin F., Parney I.F., Pavelko K.D., Johnson A.J. (2016). Improved Treatment Efficacy of Antiangiogenic Therapy when Combined with Picornavirus Vaccination in the GL261 Glioma Model. Neurotherapeutics.

[92] Yang Y., Wang C., Sun H., Jiang Z., Zhang Y., Pan Z. (2021). Apatinib prevents natural killer cell dysfunction to enhance the efficacy of anti-PD-1 immunotherapy in hepatocellular carcinoma. Cancer Gene Ther..

[93] LaGory E.L., Giaccia A.J. (2016). The ever-expanding role of HIF in tumour and stromal biology. Nature.

[94] Ni J., Wang X., Stojanovic A., Zhang Q., Wincher M., Bühler L., Arnold A., Correia M.P., Winkler M., Koch P.-S. (2020). Single-Cell RNA Sequencing of Tumor-Infiltrating NK Cells Reveals that Inhibition of Transcription Factor HIF-1α Unleashes NK Cell Activity. Immunity.

[95] Matuszewska K., Pereira M., Petrik D., Lawler J., Petrik J. (2021). Normalizing Tumor Vasculature to Reduce Hypoxia, Enhance Perfusion, and Optimize Therapy Uptake. Cancers.

[96] Fukumura D., Kloepper J., Amoozgar Z., Duda D.G., Jain R.K. (2018). Enhancing cancer immunotherapy using antiangiogenics: Opportunities and challenges. Nat. Rev. Clin. Oncol..

[97] Regulska K., Regulski M., Karolak B., Murias M., Stanisz B. (2019). Can cardiovascular drugs support cancer treatment? The rationale for drug repurposing. Drug Discov. Today.

[98] Chauhan V.P., Chen I.X., Tong R., Ng M.R., Martin J.D., Naxerova K., Wu M.W., Huang P., Boucher Y., Kohane D.S. (2019). Reprogramming the microenvironment with tumor-selective angiotensin blockers enhances cancer immunotherapy. Proc. Natl. Acad. Sci. USA.

[99] Pinter M., Jain R.K. (2017). Targeting the renin-angiotensin system to improve cancer treatment: Implications for immunotherapy. Sci. Transl. Med..

[100] Kosugi M., Miyajima A., Kikuchi E., Horiguchi Y., Murai M. (2006). Angiotensin II Type 1 Receptor Antagonist Candesartan as an Angiogenic Inhibitor in a Xenograft Model of Bladder Cancer. Clin. Cancer Res..

[101] Gelosa P., Castiglioni L., Camera M., Sironi L. (2020). Repurposing of drugs approved for cardiovascular diseases: Opportunity or mirage?. Biochem. Pharmacol..

[102] Kokolus K.M., Zhang Y., Sivik J.M., Schmeck C., Zhu J., Repasky E.A., Drabick J.J., Schell T.D. (2018). Beta blocker use correlates with better overall survival in metastatic melanoma patients and improves the efficacy of immunotherapies in mice. Oncoimmunology.

[103] Wrobel L.J., Bod L., Lengagne R., Kato M., Prévost-Blondel A., Le Gal F.-A. (2016). Propranolol induces a favourable shift of anti-tumor immunity in a murine spontaneous model of melanoma. Oncotarget.

[104] Oh M.S., Guzner A., Wainwright D.A., Mohindra N.A., Chae Y.K., Behdad A., Villaflor V.M. (2021). The Impact of Beta Blockers on Survival Outcomes in Patients with Non–small-cell Lung Cancer Treated with Immune Checkpoint Inhibitors. Clin. Lung Cancer.

[105] Pu D., Yin L., Huang L., Qin C., Zhou Y., Wu Q., Li Y., Zhou Q., Li L. (2021). Cyclooxygenase-2 Inhibitor: A Potential Combination Strategy with Immunotherapy in Cancer. Front. Oncol..

[106] Vane J., Botting R. (2003). The mechanism of action of aspirin. Thromb. Res..

[107] Law A.M.K., Valdes-Mora F., Gallego-Ortega D. (2020). Myeloid-Derived Suppressor Cells as a Therapeutic Target for Cancer. Cells.

[108] Winnicka K., Bielawski K., Bielawska A. (2006). Cardiac glycosides in cancer research and cancer therapy. Acta Pol. Pharm..

[109] Li X., Zheng J., Chen S., Meng F.-D., Ning J., Sun S.-L. (2021). Oleandrin, a cardiac glycoside, induces immunogenic cell death via the PERK/elF2α/ATF4/CHOP pathway in breast cancer. Cell Death Dis..

[110] Schneider N.F.Z., Cerella C., Simões C.M.O., Diederich M. (2017). Anticancer and Immunogenic Properties of Cardiac Glycosides. Molecules.

[111] Zhu F., Yao W., Huang Y., Chen Y., Wang Z., Cai X. (2022). Candesartan induces tumor vascular normalization to improve the efficacy of radiotherapy in the therapeutic window. Ann. Transl. Med..

[112] Keith S.W., Maio V., Arafat H.A., Alcusky M., Karagiannis T., Rabinowitz C., Lavu H., Louis D.Z. (2022). Angiotensin blockade therapy and survival in pancreatic cancer: A population study. BMC Cancer.

[113] Wei J., Zhou Z., Xu Z., Zeng S., Chen X., Wang X., Liu W., Liu M., Gong Z., Yan Y. (2019). Retrospective clinical study of renin-angiotensin system blockers in lung cancer patients with hypertension. PeerJ.

[114] Ishida J., Konishi M., Ebner N., Springer J. (2016). Repurposing of approved cardiovascular drugs. J. Transl. Med..

[115] O’rawe M., Kilmister E.J., Mantamadiotis T., Kaye A.H., Tan S.T., Wickremesekera A.C. (2021). The Renin–Angiotensin System in the Tumor Microenvironment of Glioblastoma. Cancers.

[116] Catarata M.J., Ribeiro R., Oliveira M.J., Cordeiro C.R., Medeiros R. (2020). Renin-Angiotensin System in Lung Tumor and Microenvironment Interactions. Cancers.

[117] Wadsworth B.J., Cederberg R.A., Lee C.-M., Firmino N.S., Franks S.E., Pan J., Colpo N., Lin K.-S., Benard F., Bennewith K.L. (2020). Angiotensin II type 1 receptor blocker telmisartan inhibits the development of transient hypoxia and improves tumour response to radiation. Cancer Lett..

[118] Wadsworth B.J., Lee C.-M., Urban R., Hamilton S.N., Bennewith K.L. (2021). Abstract PR-001: Angiotensin II receptor blockers modify the solid tumor microenvironment and improve radiation therapy response. Clin. Cancer Res..

[119] Stangier J., Su C., Roth W. (2000). Pharmacokinetics of Orally and Intravenously Administered Telmisartan in Healthy Young and Elderly Volunteers and in Hypertensive Patients. J. Int. Med. Res..

[120] Datta M., Coussens L.M., Nishikawa H., Hodi F.S., Jain R.K. (2019). Reprogramming the Tumor Microenvironment to Improve Immunotherapy: Emerging Strategies and Combination Therapies. Am. Soc. Clin. Oncol. Educ. Book.

[121] Gandhi S., Pandey M.R., Attwood K., Ji W., Witkiewicz A.K., Knudsen E.S., Allen C., Tario J.D., Wallace P.K., Cedeno C.D. (2021). Phase I Clinical Trial of Combination Propranolol and Pembrolizumab in Locally Advanced and Metastatic Melanoma: Safety, Tolerability, and Preliminary Evidence of Antitumor Activity. Clin. Cancer Res..

[122] Mukherjee P., Basu G.D., Tinder T.L., Subramani D.B., Bradley J.M., Arefayene M., Skaar T., De Petris G. (2009). Progression of Pancreatic Adenocarcinoma Is Significantly Impeded with a Combination of Vaccine and COX-2 Inhibition. J. Immunol..

[123] El-Fattah E.E.A. (2022). IDO/kynurenine pathway in cancer: Possible therapeutic approaches. J. Transl. Med..

[124] Müller N. (2019). COX-2 Inhibitors, Aspirin, and Other Potential Anti-Inflammatory Treatments for Psychiatric Disorders. Front. Psychiatry.

[125] Pereira A.C.A., da Silva R.J., Franco P.S., de Oliveira Gomes A., Souza G., Milian I.C.B., Ribeiro M., Rosini A.M., Guirelli P.M., Ramos E.L.P. (2019). Cyclooxygenase (COX)-2 Inhibitors Reduce *Toxoplasma gondii* Infection and Upregulate the Pro-inflammatory Immune Response in *Calomys callosus* Rodents and Human Monocyte Cell Line. Front. Microbiol..

[126] Ma S., Song W., Xu Y., Si X., Zhang Y., Tang Z., Chen X. (2020). A ROS-Responsive Aspirin Polymeric Prodrug for Modulation of Tumor Microenvironment and Cancer Immunotherapy. CCS Chem..

[127] Škubník J., Pavlíčková V., Rimpelová S. (2021). Cardiac Glycosides as Immune System Modulators. Biomolecules.

[128] Reddy D., Kumavath R., Barh D., Azevedo V., Ghosh P. (2020). Anticancer and Antiviral Properties of Cardiac Glycosides: A Review to Explore the Mechanism of Actions. Molecules.

[129] Samant R.S., Shevde L.A. (2011). Recent Advances in Anti-Angiogenic Therapy of Cancer. Oncotarget.

[130] Batchelor T.T., Gerstner E.R., Emblem K.E., Duda D.G., Kalpathy-Cramer J., Snuderl M., Ancukiewicz M., Polaskova P., Pinho M.C., Jennings D. (2013). Improved tumor oxygenation and survival in glioblastoma patients who show increased blood perfusion after cediranib and chemoradiation. Proc. Natl. Acad. Sci. USA.

[131] Wong P.P., Bodrug N., Hodivala-Dilke K.M. (2016). Exploring Novel Methods for Modulating Tumor Blood Vessels in Cancer Treatment. Curr. Biol..

[132] Simsek C., Esin E., Yalcin S. (2019). Metronomic Chemotherapy: A Systematic Review of the Literature and Clinical Experience. J. Oncol..

[133] Scharovsky O.G., Rico M.J., Mainetti L.E., Perroud H.A., Rozados V.R. (2020). Achievements and challenges in the use of metronomics for the treatment of breast cancer. Biochem. Pharmacol..

[134] Gilabert-Oriol R., Ryan G.M., Leung A.W., Firmino N.S., Bennewith K.L., Bally M.B. (2018). Liposomal Formulations to Modulate the Tumour Microenvironment and Antitumour Immune Response. Int. J. Mol. Sci..

[135] Wu J., Waxman D.J. (2018). Immunogenic chemotherapy: Dose and schedule dependence and combination with immunotherapy. Cancer Lett..

[136] Decraene B., Yang Y., De Smet F., Garg A.D., Agostinis P., De Vleeschouwer S. (2022). Immunogenic cell death and its therapeutic or prognostic potential in high-grade glioma. Genes Immun..

[137] Kaur P., Johnson A., Northcote-Smith J., Lu C., Suntharalingam K. (2020). Immunogenic Cell Death of Breast Cancer Stem Cells Induced by an Endoplasmic Reticulum-Targeting Copper(II) Complex. Chembiochem.

[138] Zhou J., Wang G., Chen Y., Wang H., Hua Y., Cai Z. (2019). Immunogenic cell death in cancer therapy: Present and emerging inducers. J. Cell. Mol. Med..

[139] Schaaf M.B., Garg A.D., Agostinis P. (2018). Defining the role of the tumor vasculature in antitumor immunity and immunotherapy. Cell Death Dis..

[140] Winkler F., Kozin S.V., Tong R.T., Chae S.-S., Booth M.F., Garkavtsev I., Xu L., Hicklin D.J., Fukumura D., di Tomaso E. (2004). Kinetics of vascular normalization by VEGFR2 blockade governs brain tumor response to radiation: Role of oxygenation, angiopoietin-1, and matrix metalloproteinases. Cancer Cell.

[141] Verma R., Foster R.E., Horgan K., Mounsey K., Nixon H., Smalle N., Hughes T.A., Carter C.R. (2016). Lymphocyte depletion and repopulation after chemotherapy for primary breast cancer. Breast Cancer Res..

[142] Li J.-Y., Chen Y.-P., Li Y.-Q., Na Liu N., Ma J. (2021). Chemotherapeutic and targeted agents can modulate the tumor microenvironment and increase the efficacy of immune checkpoint blockades. Mol. Cancer.

[143] El-Arab L.R.E., Swellam M., El Mahdy M.M. (2012). Metronomic chemotherapy in metastatic breast cancer: Impact on VEGF. J. Egypt. Natl. Cancer Inst..

[144] Krajnak S., Battista M.J., Hasenburg A., Schmidt M. (2022). Metronomic Chemotherapy for Metastatic Breast Cancer. Oncol. Res. Treat..

[145] Liu J., He M., Wang Z., Li Q., Xu B. (2022). Current Research Status of Metronomic Chemotherapy in Combination Treatment of Breast Cancer. Oncol. Res. Treat..

[146] Mancuso P., Colleoni M., Calleri A., Orlando L., Maisonneuve P., Pruneri G., Agliano A., Goldhirsch A., Shaked Y., Kerbel R.S. (2006). Circulating endothelial-cell kinetics and viability predict survival in breast cancer patients receiving metronomic chemotherapy. Blood.

[147] Cazzaniga M.E., Cordani N., Capici S., Cogliati V., Riva F., Cerrito M.G. (2021). Metronomic Chemotherapy. Cancers.

[148] Yoshimoto M., Takao S., Hirata M., Okamoto Y., Yamashita S., Kawaguchi Y., Takami M., Furusawa H., Morita S., Abe C. (2012). Metronomic oral combination chemotherapy with capecitabine and cyclophosphamide: A phase II study in patients with HER2-negative metastatic breast cancer. Cancer Chemother. Pharmacol..

[149] Patten S.G., Adamcic U., Lacombe K., Minhas K., Skowronski K., Coomber B.L. (2010). VEGFR2 heterogeneity and response to anti-angiogenic low dose metronomic cyclophosphamide treatment. BMC Cancer.

[150] Orecchioni S., Talarico G., Labanca V., Calleri A., Mancuso P., Bertolini F. (2018). Vinorelbine, cyclophosphamide and 5-FU effects on the circulating and intratumoural landscape of immune cells improve anti-PD-L1 efficacy in preclinical models of breast cancer and lymphoma. Br. J. Cancer.

[151] Webb E.R., Moreno-Vicente J., Easton A., Lanati S., Taylor M., James S., Williams E.L., English V., Penfold C., Beers S.A. (2022). Cyclophosphamide depletes tumor infiltrating T regulatory cells and combined with anti-PD-1 therapy improves survival in murine neuroblastoma. iScience.

[152] Shu Y., Weng S., Zheng S. (2020). Metronomic chemotherapy in non-small cell lung cancer (Review). Oncol. Lett..

[153] Briasoulis E., Aravantinos G., Kouvatseas G., Pappas P., Biziota E., Sainis I., Makatsoris T., Varthalitis I., Xanthakis I., Vassias A. (2013). Dose selection trial of metronomic oral vinorelbine monotherapy in patients with metastatic cancer: A hellenic cooperative oncology group clinical translational study. BMC Cancer.

[154] Camerini A., Puccetti C., Donati S., Valsuani C., Petrella M.C., Tartarelli G., Puccinelli P., Amoroso D. (2015). Metronomic oral vinorelbine as first-line treatment in elderly patients with advanced non-small cell lung cancer: Results of a phase II trial (MOVE trial). BMC Cancer.

[155] Lissoni P., Rovelli F., Malugani F., Brivio F., Fumagalli L., Gardani G. (2003). Changes in Circulating VEGF Levels in Relation to Clinical Response during Chemotherapy for Metastatic Cancer. Int. J. Biol. Markers.

[156] Orlandi P., Banchi M., Alì G., Di Desidero T., Fini E., Fontanini G., Bocci G. (2021). Active metronomic vinorelbine schedules decrease plasma interleukin-2 levels in mice with Lewis lung carcinoma. J. Chemother..

[157] Katsaounis P., Kotsakis A., Agelaki S., Kontopodis E., Agelidou A., Kentepozidis N., Vamvakas L., Christopoulou A., Karachaliou N., Hatzidaki D. (2015). Cisplatin in combination with metronomic vinorelbine as front-line treatment in advanced non-small cell lung cancer: A multicenter phase II study of the Hellenic Oncology Research Group (HORG). Cancer Chemother. Pharmacol..

[158] Correale P., Remondo C., Carbone S.F., Ricci V., Migali C., Martellucci I., Licchetta A., Addeo R., Volterrani L., Gotti G. (2010). Dose/dense metronomic chemotherapy with fractioned cisplatin and oral daily etoposide enhances the anti-angiogenic effects of bevacizumab and has strong anti-tumor activity in advanced non-small-cell-lung cancer patients. Cancer Biol. Ther..

[159] Skavatsou E., Semitekolou M., Morianos I., Karampelas T., Lougiakis N., Xanthou G., Tamvakopoulos C. (2021). Immunotherapy Combined with Metronomic Dosing: An Effective Approach for the Treatment of NSCLC. Cancers.

[160] Zhu N., Qin R., Zhang Q., Fu S., Liu S., Chen Y., Fan J., Han Y. (2018). Efficacy of granulocyte-macrophage colony-stimulating factor combined with metronomic paclitaxel in the treatment of Lewis lung carcinoma transplanted in mice. Oncotarget.

[161] Baert T., Ferrero A., Sehouli J., O’Donnell D., González-Martín A., Joly F., van der Velden J., Blecharz P., Tan D., Querleu D. (2021). The systemic treatment of recurrent ovarian cancer revisited. Ann. Oncol..

[162] Sharma A., Malik P., Khurana S., Kumar S., Bhatla N., Ray M.D., Kumar L. (2019). Oral metronomic chemotherapy for recurrent & refractory epithelial ovarian cancer: A retrospective analysis. Indian J. Med. Res..

[163] Garcia A.A., Hirte H., Fleming G., Yang D., Tsao-Wei D.D., Roman L., Groshen S., Swenson S., Markland F., Gandara D. (2008). Phase II Clinical Trial of Bevacizumab and Low-Dose Metronomic Oral Cyclophosphamide in Recurrent Ovarian Cancer: A Trial of the California, Chicago, and Princess Margaret Hospital Phase II Consortia. J. Clin. Oncol..

[164] Burger R.A., Brady M.F., Bookman M.A., Fleming G.F., Monk B.J., Huang H., Mannel R.S., Homesley H.D., Fowler J., Greer B.E. (2011). Incorporation of Bevacizumab in the Primary Treatment of Ovarian Cancer. N. Engl. J. Med..

[165] De Boo L.W., Vulink A.J.E., Bos M.E.M.M. (2017). Metronomic cyclophosphamide-induced long-term remission after recurrent high-grade serous ovarian cancer: A case study. Mol. Clin. Oncol..

[166] Malik P.S., Raina V., André N. (2014). Metronomics as Maintenance Treatment in Oncology: Time for Chemo-Switch. Front. Oncol..

[167] Zsiros E., Lynam S., Attwood K.M., Wang C., Chilakapati S., Gomez E.C., Liu S., Akers S., Lele S., Frederick P.J. (2021). Efficacy and Safety of Pembrolizumab in Combination with Bevacizumab and Oral Metronomic Cyclophosphamide in the Treatment of Recurrent Ovarian Cancer. JAMA Oncol..

[168] Tamura R., Miyoshi H., Yoshida K., Okano H., Toda M. (2021). Recent progress in the research of suicide gene therapy for malignant glioma. Neurosurg. Rev..

[169] Ghiaseddin A.P., Shin D., Melnick K., Tran D.D. (2020). Tumor Treating Fields in the Management of Patients with Malignant Gliomas. Curr. Treat. Options Oncol..

[170] Sousa M.J., Magalhães J., Basto R., Costa C., Pego A., Sousa G. (2021). P14.90 Survival outcomes and prognostic factors in glioblastoma patients treated with radiotherapy plus concomitant and adjuvant temozolomide—Real-world study. J. Neuro-Oncol..

[171] Hotchkiss K.M., Sampson J.H. (2021). Temozolomide treatment outcomes and immunotherapy efficacy in brain tumor. J. Neuro-Oncol..

[172] Perry J.R., Rizek P., Cashman R., Morrison M., Morrison T. (2008). Temozolomide rechallenge in recurrent malignant glioma by using a continuous temozolomide schedule. Cancer.

[173] Reardon D.A., Desjardins A., Vredenburgh J.J., Gururangan S., Sampson J.H., Sathornsumetee S., McLendon R.E., Herndon J.E., Marcello J.E., Norfleet J. (2009). Metronomic chemotherapy with daily, oral etoposide plus bevacizumab for recurrent malignant glioma: A phase II study. Br. J. Cancer.

[174] Reardon D.A., Desjardins A., Peters K., Gururangan S., Sampson J., Rich J.N., McLendon R., Herndon J.E., Marcello J., Threatt S. (2011). Phase II study of metronomic chemotherapy with bevacizumab for recurrent glioblastoma after progression on bevacizumab therapy. J. Neuro-Oncol..

[175] Amoozgar Z., Kloepper J., Ren J., Tay R.E., Kazer S.W., Kiner E., Krishnan S., Posada J.M., Ghosh M., Mamessier E. (2021). Targeting Treg cells with GITR activation alleviates resistance to immunotherapy in murine glioblastomas. Nat. Commun..

[176] Datta M., Chatterjee S., Perez E.M., Gritsch S., Roberge S., Duquette M., Chen I.X., Naxerova K., Kumar A.S., Ghosh M. (2023). Losartan controls immune checkpoint blocker-induced edema and improves survival in glioblastoma mouse models. Proc. Natl. Acad. Sci. USA.

[177] Amoozgar Z., Ren J., Wang N., Andersson P., Ferraro G., Rajan S., Lei P., Subudhi S., Kawaguchi K., Tay R.E. (2022). Combined blockade of VEGF, Angiopoietin-2, and PD1 reprograms glioblastoma endothelial cells into quasi-antigen-presenting cells. bioRxiv.

[178] Mathew E.N., Berry B.C., Yang H.W., Carroll R.S., Johnson M.D. (2022). Delivering Therapeutics to Glioblastoma: Overcoming Biological Constraints. Int. J. Mol. Sci..

[179] Walter I., Schulz U., Vogelhuber M., Wiedmann K., Endlicher E., Klebl F., Andreesen R., Herr W., Ghibelli L., Hackl C. (2017). Communicative reprogramming non-curative hepatocellular carcinoma with low-dose metronomic chemotherapy, COX-2 inhibitor and PPAR-gamma agonist: A phase II trial. Med. Oncol..

[180] Wysocki P.J., Lubas M.T., Wysocka M.L. (2022). Metronomic Chemotherapy in Prostate Cancer. J. Clin. Med..

[181] Jiang L., Ping L., Yan H., Yang X., He Q., Xu Z., Luo P. (2020). Cardiovascular toxicity induced by anti-VEGF/VEGFR agents: A special focus on definitions, diagnoses, mechanisms and management. Expert Opin. Drug Metab. Toxicol..

[182] Liang Q., Zhou L., Li Y., Liu J., Liu Y. (2021). Nano drug delivery system reconstruct tumour vasculature for the tumour vascular normalisation. J. Drug Target..

[183] Mattheolabakis G., Mikelis C.M. (2019). Nanoparticle Delivery and Tumor Vascular Normalization: The Chicken or The Egg?. Front. Oncol..

[184] Bulbake U., Doppalapudi S., Kommineni N., Khan W. (2017). Liposomal Formulations in Clinical Use: An Updated Review. Pharmaceutics.

[185] Allen T.M., Cullis P.R. (2004). Drug Delivery Systems: Entering the Mainstream. Science.

[186] Gabizon A.A. (1995). Liposome circulation time and tumor targeting: Implications for cancer chemotherapy. Adv. Drug Deliv. Rev..

[187] Litzinger D.C., Buiting A.M., van Rooijen N., Huang L. (1994). Effect of liposome size on the circulation time and intraorgan distribution of amphipathic poly(ethylene glycol)-containing liposomes. (BBA)—Biomembr..

[188] Li S.-D., Huang L. (2008). Pharmacokinetics and Biodistribution of Nanoparticles. Mol. Pharm..

[189] Hashizume H., Baluk P., Morikawa S., McLean J.W., Thurston G., Roberge S., Jain R.K., McDonald D.M. (2000). Openings between Defective Endothelial Cells Explain Tumor Vessel Leakiness. Am. J. Pathol..

[190] Guyon J., Chapouly C., Andrique L., Bikfalvi A., Daubon T. (2021). The Normal and Brain Tumor Vasculature: Morphological and Functional Characteristics and Therapeutic Targeting. Front. Physiol..

[191] Nagy J., Chang S.-H., Shih S.-C., Dvorak A., Dvorak H. (2010). Heterogeneity of the Tumor Vasculature. Semin. Thromb. Hemost..

[192] Wei X., Meel M.H., Breur M., Bugiani M., Hulleman E., Phoenix T.N. (2021). Defining tumor-associated vascular heterogeneity in pediatric high-grade and diffuse midline gliomas. Acta Neuropathol. Commun..

[193] Stapleton S., Milosevic M., Allen C., Zheng J., Dunne M., Yeung I., Jaffray D.A. (2013). A Mathematical Model of the Enhanced Permeability and Retention Effect for Liposome Transport in Solid Tumors. PLoS ONE.

[194] Fang J., Islam W., Maeda H. (2020). Exploiting the dynamics of the EPR effect and strategies to improve the therapeutic effects of nanomedicines by using EPR effect enhancers. Adv. Drug Deliv. Rev..

[195] Kamaly N., Yameen B., Wu J., Farokhzad O.C. (2016). Degradable Controlled-Release Polymers and Polymeric Nanoparticles: Mechanisms of Controlling Drug Release. Chem. Rev..

[196] Jacobson G.B., Shinde R., Contag C.H., Zare R.N. (2008). Sustained Release of Drugs Dispersed in Polymer Nanoparticles. Angew. Chem. Int. Ed..

[197] Kumar D., Archana, Niranjan A.K. (2022). A Comprehensive Review on Sustained Release Matrix Drug Delivery System. J. Drug Deliv. Ther..

[198] Karumanchi D.K., Skrypai Y., Thomas A., Gaillard E.R. (2018). Rational design of liposomes for sustained release drug delivery of bevacizumab to treat ocular angiogenesis. J. Drug Deliv. Sci. Technol..

[199] Vasantha J., Kannan G., Goud T., Palani T., Vanitha R., Anitha R., Priya J. (2011). Pharmacokinetic Evaluation of Paclitaxel in South Indian Cancer Patients: A Prospective Study. J. Young Pharm..

[200] Emmenegger U., Shaked Y., Man S., Bocci G., Spasojevic I., Francia G., Kouri A., Coke R., Cruz-Munoz W., Ludeman S.M. (2007). Pharmacodynamic and pharmacokinetic study of chronic low-dose metronomic cyclophosphamide therapy in mice. Mol. Cancer Ther..

[201] Bocci G., Kerbel R.S. (2016). Pharmacokinetics of metronomic chemotherapy: A neglected but crucial aspect. Nat. Rev. Clin. Oncol..

[202] Gabizon A., Ohana P., Amitay Y., Gorin J., Tzemach D., Mak L., Shmeeda H. (2021). Liposome co-encapsulation of anti-cancer agents for pharmacological optimization of nanomedicine-based combination chemotherapy. Cancer Drug Resist..

[203] Blair H.A. (2018). Daunorubicin/Cytarabine Liposome: A Review in Acute Myeloid Leukaemia. Drugs.

[204] Mayer L.D., Harasym T.O., Tardi P.G., Harasym N.L., Shew C.R., Johnstone S.A., Ramsay E.C., Bally M.B., Janoff A.S. (2006). Ratiometric dosing of anticancer drug combinations: Controlling drug ratios after systemic administration regulates therapeutic activity in tumor-bearing mice. Mol. Cancer Ther..

[205] Ramsay E.C., Dos Santos N., Dragowska W.H., Laskin J.J., Bally M. (2005). The Formulation of Lipid-Based Nanotechnologies for the Delivery of Fixed Dose Anticancer Drug Combinations. Curr. Drug Deliv..

[206] Neijzen R., Wong M.Q., Gill N., Wang H., Karim T., Anantha M., Strutt D., Waterhouse D., Bally M.B., Tai I.T. (2015). Irinophore C™, a lipid nanoparticulate formulation of irinotecan, improves vascular function, increases the delivery of sequentially administered 5-FU in HT-29 tumors, and controls tumor growth in patient derived xenografts of colon cancer. J. Control. Release.

[207] Gu Z., Da Silva C.G., Van der Maaden K., Ossendorp F., Cruz L.J. (2020). Liposome-Based Drug Delivery Systems in Cancer Immunotherapy. Pharmaceutics.

[208] Verreault M., Strutt D., Masin D., Anantha M., Yung A., Kozlowski P., Waterhouse D., Bally M.B., Yapp D.T. (2011). Vascular normalization in orthotopic glioblastoma following intravenous treatment with lipid-based nanoparticulate formulations of irinotecan (Irinophore C™), doxorubicin (Caelyx^®^) or vincristine. BMC Cancer.

[209] Chaudhuri T.R., Arnold R.D., Yang J., Turowski S.G., Qu Y., Spernyak J.A., Mazurchuk R., Mager D.E., Straubinger R.M. (2012). Mechanisms of Tumor Vascular Priming by a Nanoparticulate Doxorubicin Formulation. Pharm. Res..

[210] Rios-Doria J., Durham N., Wetzel L., Rothstein R., Chesebrough J., Holoweckyj N., Zhao W., Leow C.C., Hollingsworth R. (2015). Doxil Synergizes with Cancer Immunotherapies to Enhance Antitumor Responses in Syngeneic Mouse Models. Neoplasia.

[211] Mpekris F., Panagi M., Voutouri C., Martin J.D., Samuel R., Takahashi S., Gotohda N., Suzuki T., Papageorgis P., Demetriou P. (2020). Normalizing the Microenvironment Overcomes Vessel Compression and Resistance to Nano-immunotherapy in Breast Cancer Lung Metastasis. Adv. Sci..

[212] Ngo W., Ahmed S., Blackadar C., Bussin B., Ji Q., Mladjenovic S.M., Sepahi Z., Chan W.C. (2022). Why nanoparticles prefer liver macrophage cell uptake in vivo. Adv. Drug Deliv. Rev..

[213] Liu C.-Y., Tsai T.-H., Huang Y.-C., Shieh H.-R., Liao H.-F., Chen Y.-J. (2012). Differential immunomodulating effects of pegylated liposomal doxorubicin nanoparticles on human macrophages. J. Nanosci. Nanotechnol..

[214] Strieth S., Eichhorn M.E., Werner A., Sauer B., Teifel M., Michaelis U., Berghaus A., Dellian M. (2008). Paclitaxel Encapsulated in Cationic Liposomes Increases Tumor Microvessel Leakiness and Improves Therapeutic Efficacy in Combination with Cisplatin. Clin. Cancer Res..

[215] Strieth S., Eichhorn M.E., Sauer B., Schulze B., Teifel M., Michaelis U., Dellian M. (2004). Neovascular targeting chemotherapy: Encapsulation of paclitaxel in cationic liposomes impairs functional tumor microvasculature. Int. J. Cancer.

[216] Bocci G., Di Paolo A., Danesi R. (2013). The pharmacological bases of the antiangiogenic activity of paclitaxel. Angiogenesis.

[217] Huo M., Wang H., Zhang Y., Cai H., Zhang P., Li L., Zhou J., Yin T. (2020). Co-delivery of silybin and paclitaxel by dextran-based nanoparticles for effective anti-tumor treatment through chemotherapy sensitization and microenvironment modulation. J. Control. Release.

[218] Kim J.Y., Kim Y.-M. (2019). Tumor endothelial cells as a potential target of metronomic chemotherapy. Arch. Pharmacal Res..

[219] Huang D., Sun L., Huang L., Chen Y. (2021). Nanodrug Delivery Systems Modulate Tumor Vessels to Increase the Enhanced Permeability and Retention Effect. J. Pers. Med..

[220] Cai X.-J., Fei W., Xu Y.-Y., Xu H., Yang G.-Y., Cao J.-W., Ni J.-J., Tao K., Wang Z. (2020). Liposome-Encapsulated Zoledronate Favors Tumor Vascular Normalization and Enhances Anticancer Efficacy of Cisplatin. AAPS PharmSciTech.

[221] Luput L., Sesarman A., Porfire A., Achim M., Muntean D., Casian T., Patras L., Rauca V.F., Drotar D.M., Stejerean I. (2020). Liposomal simvastatin sensitizes C26 murine colon carcinoma to the antitumor effects of liposomal 5-fluorouracil in vivo. Cancer Sci..

[222] Li Y., Du B., Cheng G. (2020). Reshaping Tumor Blood Vessels to Enhance Drug Penetration with a Multistrategy Synergistic Nanosystem. Mol. Pharm..

[223] Li Y., Wang J., Gao Y., Zhu J., Wientjes M.G., Au J.L.-S. (2011). Relationships between Liposome Properties, Cell Membrane Binding, Intracellular Processing, and Intracellular Bioavailability. AAPS J..

[224] Liu X., Jiang J., Liao Y., Tang I., Zheng E., Qiu W., Lin M., Wang X., Ji Y., Mei K. (2021). Combination Chemo-Immunotherapy for Pancreatic Cancer Using the Immunogenic Effects of an Irinotecan Silicasome Nanocarrier Plus Anti-PD-1. Adv. Sci..

[225] Piazzini V., Landucci E., Graverini G., Pellegrini-Giampietro D.E., Bilia A.R., Bergonzi M.C. (2018). Stealth and Cationic Nanoliposomes as Drug Delivery Systems to Increase Andrographolide BBB Permeability. Pharmaceutics.

[226] Sakurai Y., Hada T., Yamamoto S., Kato A., Mizumura W., Harashima H. (2016). Remodeling of the Extracellular Matrix by Endothelial Cell-Targeting siRNA Improves the EPR-Based Delivery of 100 nm Particles. Mol. Ther..

[227] Tabernero J., Shapiro G.I., Lorusso P.M., Cervantes A., Schwartz G.K., Weiss G.J., Paz-Ares L., Cho D.C., Infante J.R., Alsina M. (2013). First-in-Humans Trial of an RNA Interference Therapeutic Targeting VEGF and KSP in Cancer Patients with Liver Involvement. Cancer Discov..

[228] Qi L., Xing L., Wei X., Song S. (2014). Effects of VEGF suppression by small hairpin RNA interference combined with radiotherapy on the growth of cervical cancer. Genet. Mol. Res..

[229] Jenkins R.W., Barbie D.A., Flaherty K.T. (2018). Mechanisms of resistance to immune checkpoint inhibitors. Br. J. Cancer.

[230] Sim M.J.W., Sun P.D. (2022). T Cell Recognition of Tumor Neoantigens and Insights into T Cell Immunotherapy. Front. Immunol..

[231] Hos B.J., Camps M.G., Bulk J.V.D., Tondini E., Ende T.C.V.D., Ruano D., Franken K., Janssen G.M., de Ru A.H., Filippov D.V. (2019). Identification of a neo-epitope dominating endogenous CD8 T cell responses to MC-38 colorectal cancer. Oncoimmunology.

[232] Roviello G., Catalano M., Santi R., Palmieri V.E., Vannini G., Galli I.C., Buttitta E., Villari D., Rossi V., Nesi G. (2021). Immune Checkpoint Inhibitors in Urothelial Bladder Cancer: State of the Art and Future Perspectives. Cancers.

[233] Marofi F., Motavalli R., Safonov V.A., Thangavelu L., Yumashev A.V., Alexander M., Shomali N., Chartrand M.S., Pathak Y., Jarahian M. (2021). CAR T cells in solid tumors: Challenges and opportunities. Stem Cell Res. Ther..

[234] Newick K., O’Brien S., Moon E., Albelda S.M. (2017). CAR T Cell Therapy for Solid Tumors. Annu. Rev. Med..

[235] Ba E.A.Y., Shi Y., Jiang W., Feng J., Cheng Y., Xiao L., Zhang Q., Qiu W., Xu B., Xu R. (2020). Current management of chemotherapy-induced neutropenia in adults: Key points and new challenges. Cancer Biol. Med..

[236] Das R.K., O’connor R.S., Grupp S.A., Barrett D.M. (2020). Lingering effects of chemotherapy on mature T cells impair proliferation. Blood Adv..

[237] Watson N., Al-Samkari H. (2021). Thrombotic and bleeding risk of angiogenesis inhibitors in patients with and without malignancy. J. Thromb. Haemost..

[238] Elice F., Rodeghiero F. (2012). Side effects of anti-angiogenic drugs. Thromb. Res..

